# A global functional analysis of missense mutations reveals two major hotspots in the *PALB2* tumor suppressor

**DOI:** 10.1093/nar/gkz780

**Published:** 2019-10-05

**Authors:** Amélie Rodrigue, Guillaume Margaillan, Thiago Torres Gomes, Yan Coulombe, Gemma Montalban, Simone da Costa e Silva Carvalho, Larissa Milano, Mandy Ducy, Giuliana De-Gregoriis, Graham Dellaire, Wilson Araújo da Silva Jr, Alvaro N Monteiro, Marcelo A Carvalho, Jacques Simard, Jean-Yves Masson

**Affiliations:** 1 CHU de Québec-Université Laval, Oncology Division, 9 McMahon, Québec City, QC G1R 3S3, Canada; 2 Department of Molecular Biology, Medical Biochemistry and Pathology; Laval University Cancer Research Center, Québec City, QC G1V 0A6, Canada; 3 CHU de Québec-Université Laval Research Center, Genomics Center, Québec City, QC, Canada; 4 Instituto Nacional de Câncer, Centro de Pesquisa, Programa de Pesquisa Clínica, Rio de Janeiro, Brazil; 5 Instituto Federal do Rio de Janeiro, Laboratório de Genética Molecular, Maracanã, Rio de Janeiro, Brazil; 6 Department of Genetics at Ribeirão Preto Medical School, University of São Paulo; Center for Cell-Based Therapy (CEPID/FAPESP); National Institute of Science and Technology in Stem Cell and Cell Therapy (INCTC/CNPq), Ribeirão Preto, SP, Brazil; 7 Department of Pathology, Dalhousie University, Halifax, NS, Canada; 8 H. Lee Moffitt Cancer Center & Research Institute, Tampa, FL, USA

## Abstract

While biallelic mutations in the *PALB2* tumor suppressor cause Fanconi anemia subtype FA-N, monoallelic mutations predispose to breast and familial pancreatic cancer. Although hundreds of missense variants in *PALB2* have been identified in patients to date, only a few have clear functional and clinical relevance. Herein, we investigate the effects of 44 *PALB2* variants of uncertain significance found in breast cancer patients and provide detailed analysis by systematic functional assays. Our comprehensive functional analysis reveals two hotspots for potentially deleterious variations within PALB2, one at each terminus. PALB2 N-terminus variants p.P8L [c.23C>T], p.Y28C [c.83A>G], and p.R37H [c.110G>A] compromised PALB2-mediated homologous recombination. At the C-terminus, PALB2 variants p.L947F [c.2841G>T], p.L947S [c.2840T>C], and most strikingly p.T1030I [c.3089C>T] and p.W1140G [c.3418T>C], stood out with pronounced PARP inhibitor sensitivity and cytoplasmic accumulation in addition to marked defects in recruitment to DNA damage sites, interaction with BRCA2 and homologous recombination. Altogether, our findings show that a combination of functional assays is necessary to assess the impact of germline missense variants on PALB2 function, in order to guide proper classification of their deleteriousness.

## INTRODUCTION

PALB2 (partner and localizer of BRCA2) is a crucial and versatile contributor to genome integrity maintenance and tumorigenesis suppression. While germline biallelic mutations in *PALB2* give rise to Fanconi anemia subtype FA-N (1,2), monoallelic mutations predispose to breast and familial pancreatic cancer (3–6). First identified as a binding partner and localizer of the breast cancer protein BRCA2 (7), PALB2 came to be commonly known as an essential player in the repair of DNA double-strand breaks (DSBs) by homologous recombination (HR). HR is a high-fidelity pathway that prevails in the S/G2 phase of the cell cycle, when an intact sister chromatid is available as template for error-free DSB repair. HR deficiency forces cells to rely on mutagenic DSB repair pathways for survival, leading to genomic instability and tumorigenesis (8–10). During HR, PALB2 acts as an essential bridge between BRCA1 and BRCA2 (11–13) that promotes the recruitment of the RAD51 recombinase to DNA damage sites and its assembly into nucleofilaments to initiate DSB repair (14,15). Similar to what has been described for BRCA2, loss of functional PALB2 is synthetic lethal with poly(ADP-ribose) polymerase (PARP) inhibitors, an innovative class of anti-cancer drugs (14,16–20). Although its entire spectrum of activities has yet to be discovered, PALB2 has been also linked to recombination-dependent DNA synthesis at blocked replication forks (21), cellular redox homeostasis regulation (22) and protection of active genes during DNA replication (23) via interaction with polymerase (Pol η), KEAP1 and MRG15, respectively.

Structurally, PALB2 is a 1186-amino acid protein (130 kDa), encoded by a gene located on chromosome 16p12 and consisting of 13 exons (7), that presents various protein domains. Regulation of PALB2 functions in HR has been shown to involve several of these protein domains as well as modifications, including self-interaction, phosphorylation, and ubiquitylation, and is cell cycle-dependent (6,24). In its amino-terminal region, PALB2 bears a coiled-coil domain (aa 9–44), which mediates its oligomerization (25,26) and interaction with BRCA1 (11–13). Upon damage, PALB2 is thought to switch from a low activity oligomer to a protein complex composed of BRCA1 and BRCA2 (25). The interaction with BRCA1 is regulated by phosphorylation events involving ATM/ATR and CDKs (27) and promotes the accumulation of PALB2 to DNA damage sites, which in turn facilitates the recruitment of BRCA2 and RAD51 to promote HR (11,12) in S/G2. The PALB2-BRCA1 interaction is disrupted by KEAP1-dependent ubiquitylation of PALB2 in G1 to avoid the deleterious outcomes of untimely HR (28). PALB2 N-terminal region also provides a site for interaction with KEAP1 (22) and the RAD51 recombinase (14,15).

PALB2 C-terminal presents a WD40 domain (aa 859–1186) that folds into a seven-bladed ß-propeller structure and supports binding to BRCA2 (7,29), RAD51 (14,15), RAD51C (30), RNF168 (31), and Pol η (21). This structure is of great importance for PALB2 stability, as loss of the last four codons by the Y1183X mutation is sufficient to leave the protein incompletely folded and vulnerable to degradation (1,29). Furthermore, the PALB2 WD40 domain hides a nuclear export signal (NES), buried within the propeller structure (aa 928–945), which can be exposed by cancer-associated truncations, such as W1038X, resulting in protein mislocalization to the cytoplasm and faulty functions that could drive to genetic instability (32). In the center of the protein lies an evolutionarily conserved chromatin-association motif (ChAM) (aa 395–446) shown to mediate PALB2 chromatin association and DNA repair function (33) and a MRG15-binding domain (aa 611–737) involved in tethering PALB2 to damage and undamaged chromatin (23,34,35).

To date, most pathogenic *PALB2* mutations reported in breast cancer (BC) patients are truncating mutations distributed throughout its coding region (36,37). Loss of the entire WD40 domain or only part of it leads to particularly severe HR deficiency (2,32,38,39). Unsurprisingly, *PALB2* truncating mutations have been tied to increased risks of developing the disease with lifetime risks of breast cancer of 24–54%, depending on family history of breast cancer (40). With the advent of genetic testing in clinical settings, a large number of sequence alterations in *PALB2*, mostly missense variations, have been uncovered. Among these missense variants, only a few have been fully or even partially characterized. Whether or not these missense variants are associated with increased BC risks and HR deficit remains, however, unknown for the most part, posing a challenge for genetic counselling. Recently, coiled-coil domain variants p.Y28C and p.L35P have been shown to weaken PALB2 self-interaction and abrogate PALB2-BRCA1 interaction (41). While p.L35P demonstrates complete HR impairment, loss of RAD51 foci formation and sensitivity to the PARP inhibitor (PARPi) olaparib, p.Y28C shows an intermediate phenotype, with a 65% reduction in HR activity. HR activity was decreased ∼20% for p.K18R and p.R37H variants while being unaffected for p.K30N (41). The p.L939W, p.T1030I and p.L1143P missense variants of PALB2 WD40 domain have been associated with altered binding patterns to RAD51C, RAD51 and BRCA2 (30). While p.T1030I was found unstable and most likely to be degraded, the p.L939W and p.L1143P mutants displayed a slight decrease in HR. However, this latter result regarding p.L939W was not corroborated in a more recent study (42). More recently, two missense variants in either BRCA2 (p.W31G) or PALB2 (p.P1088S) were suggested to be pathogenic based on the abrogation of BRCA2-PALB2 interaction (43). Nevertheless, p.L35P remains the sole missense variant in *PALB2* truly considered deleterious so far.

Here, we sought to establish the landscape of HR functionality and vulnerabilities to PARP inhibitors of a list of selected missense variants in *PALB2* found in BC patients. Our results demonstrate that there are two major hotspots for missense mutations affecting PALB2; one in the N-terminus and the other in the C-terminus. Moreover, the systematic functional assays presented will aid in variant assessment and its associated clinical and therapeutic management.

## MATERIALS AND METHODS

### Constructs and siRNA

The *GAL4DBD-BRCA1* wild-type (aa 1315–1863) fusion construct was previously generated (44) and *GAL4DBD-BRCA2* wild-type (aa 1–60) was kindly provided by Dr. Bing Xia (7). The *VP16 AD-PALB2* N-terminal wild-type (aa 1–319) construct was generated by amplification of the coding sequence using a previously generated PALB2 construct as template (44), while the *VP16 AD-PALB2* C-terminal wild-type construct (aa 859–1186) was obtained using a normal human leukocyte cDNA as template; both products were cloned into the pVP16 vector (Clontech) in *Eco*RI/*Bam*HI sites, downstream of the *VP16 AD* cassette. *PALB2* variants were generated using the QuickChange II Site-Directed Mutagenesis Kit (Agilent) or by overlap extension site-directed mutagenesis as described by Ho *et al.* (45), followed by cloning into the pVP16 vector using *Eco*RI/*Bam*HI restriction sites. All constructs were confirmed by sequencing. Primers are listed in Supplemental Table S4. For *in cellulo* experiments with HeLa and U2OS cells, the variants were obtained via site-directed mutagenesis on a pEYFP-C1-PALB2 vector, previously modified to be resistant to PALB2 siRNA using the Q5 Site-Directed Mutagenesis Kit with gatCTTATTGTTCTACCAGGAAAATC (forward) and ttccTCTAAGTCCTCCATTTCTG (reverse) as primers. Primers are listed in Supplemental Table S5. The siRNA target sequence used to silence PALB2 was CUUAGAAGAGGACCUUAUU and the non-specific siRNA used as control was UUCGAACGUGUCACGUCAA. For the CRISPR-LMNA HDR assays (27), we used pX330-LMNAgRNA2 (46) and a mRuby2-tagged donor instead of a mClover-LMNA donor.

### Cell lines

U2OS osteosarcoma cells (HTB-96) were purchased from American Type Culture Collection (ATCC) and maintained in McCoy's 5A supplemented with 10% Fetal Bovine Serum (FBS) and 1% penicillin/streptomycin (P/S). HEK293FT were purchased from Invitrogen and maintained in DMEM supplemented with 10% FBS. HeLa cells were authenticated using Short Tandem Repeat (STR) analysis by ATCC and maintained in DMEM supplemented with 10% FBS and 1% P/S. All cell lines were grown at 37°C, 5% CO_2_, and routinely tested to be mycoplasma free.

### Mammalian two-hybrid assay

Mammalian two-hybrid assay was conducted using the Dual-Luciferase Reporter Assay System (Promega). In brief, *PALB2* N- or C-terminal construct (wild-type or variants) was co-transfected using Polyethylenimine (Polysciences Inc.) into HEK293FT cells, together with *GAL4DBD-BRCA1* or *GAL4DBD-BRCA2*, the pG5Luc reporter vector and the pGR-TK internal control. The VP16 AD–PALB2 fusion protein acts as the prey protein in this system. Both GAL4DBD and VP16AD fusion constructs contain a Nuclear Localization Signal (NLS). When the BRCA1–PALB2 protein–protein interaction occurs, the transcriptional activity is enhanced above the levels observed for the BRCA1 or BRCA2 construct alone due to transcriptional activation mediated by the VP16 AD fused to the PALB2 protein. The reporter assay is performed 24 h post-transfection. The PALB2 L21A and A1025R variants were used as negative controls as previously reported (13,29). Results were reported in bar graphs depicting the mean percentage luciferase activity ± SEM of 3 independent experiments.

### Protein extraction and immunoblotting

HEK293FT cells transfected with the indicated plasmid constructs were lysed in mild-RIPA buffer supplemented with Protease and Phosphatase Inhibitors Cocktails (Merck). Immunoblotting analysis was performed 24 h post-transfection using anti-VP16 (1:50; Santa Cruz Biotechnology, #sc-7545; or 1:1000; Abcam, #ab4808) or anti-GAL4 (DBD) (1:500; Santa Cruz Biotechnology, #sc-510) antibodies. Total soluble protein extracts from HeLa and U2OS cells and immunoblotting were performed as described in Castroviejo-Bermejo *et al.* (47). For PALB2 detection, a polyclonal antibody was raised in rabbit and used at a 1:5000 dilution. Mouse monoclonal anti-alpha tubulin (1:200 000; Abcam, #ab7291) served as the loading control. Horseradish peroxidase-conjugated anti-rabbit IgG or anti-mouse (1:10 000; Jackson ImmunoResearch) were used as secondary antibodies.

### Olaparib sensitivity assay

For the sensitivity assay in HeLa, 240 000 cells were seeded into one well of a six-well plate before being transfected 6–8 h later with 50 nM control or PALB2 siRNA using Lipofectamine RNAiMAX (Invitrogen). The next morning, cells were complemented with 800 ng of the peYFP-C1 empty vector or the indicated siRNA-resistant YFP-tagged PALB2 construct using Lipofectamine 2000 (Invitrogen) for 24 h and then seeded in triplicates into a Corning 3603 black-sided clear bottom 96-well microplate at a density of 3000 cells per well. The remaining cells were kept and stored at −80°C until processed for protein extraction and immunoblotting as described above. Once attached to the plate, cells were exposed to different concentrations of olaparib (Selleckchem, #S1060) ranging from 0 (DMSO) to 2.5 μM. After 3 days of treatment, nuclei were stained with Hoechst 33342 (Invitrogen) at 10 μg/ml in media for 45 min at 37°C. Images of entire wells were acquired at 4x with a Cytation 5 Cell Imaging Multi-Mode Reader followed by quantification of Hoechst-stained nuclei with the Gen5 Data Analysis Software v3.03 (BioTek Instruments). Cell viability was expressed as percentage of survival in olaparib-treated cells relative to vehicle (DMSO)-treated cells. Results represent the mean ± SD of at least 3 independent experiments, each performed in triplicate. For the sensitivity assay in U2OS, transfection steps were performed as described in the section on the CRISPR-LMNA HDR assay. Then cells were plated in 96-well microplates at 2000 cells per well 24 h post-nucleofection and submitted to olaparib treatment for 5 days.

### Live-cell microscopy and laser micro-irradiation

Live-cell imaging and micro-irradiation experiments were carried out with a Leica TCS SP5 II confocal microscope driven by Leica LAS AF software using a 63×/1.4 oil immersion objective. The microscope was equipped with an environmental chamber set to 37°C and 5% CO_2_. Briefly, HeLa cells seeded onto 35-mm fluorodishes (World Precision Instruments, Inc.) were transfected with 800 ng peYFP-C1-PALB2 WT or variant constructs using Effectene transfection reagent (Qiagen). The next day, cells were micro-irradiated in the nucleus for 200 ms using a 405 nm UV-laser at the following settings: format 512 × 512 pixels, scan speed 100 Hz, mode bidirectional, zoom 2×. To monitor the recruitment of YFP-PALB2 to laser-induced DNA damage sites, cells were micro-irradiated and imaged every 30 s for 15 min, after which fluorescence intensity of YFP-PALB2 at DNA damage sites relative to an unirradiated nuclear area was quantified and plotted over time. Kinetic curves were obtained by averaging the relative fluorescence intensity of cells displaying positive recruitment (total *n* > 60 cells) and error bars show the SEM. We considered positive only cells whose relative fluorescence intensity increased over the basal 100% after irradiation. Quantification of YFP-PALB2 subcellular localization was determined from live-cell microscopy images captured prior to micro-irradiation (total *n*>120 cells). All results are from at least 3 independent experiments.

### RAD51 foci assay

HeLa cells were seeded on glass coverslips in 6-well plates at 225 000 cells per well. Knockdown of PALB2 was performed 18 h later with 50 nM PALB2 siRNA using Lipofectamine RNAiMAX (Invitrogen). After 5 h, cells were subjected to double thymidine block. Briefly, cells were treated with 2 mM thymidine for 18 h and release into fresh media for 9 h. Complementation using 800 ng of the peYFP-C1 empty vector or the indicated siRNA-resistant YFP-PALB2 construct was carried out with Lipofectamine 2000 during that release time. Then, cells were treated with 2 mM thymidine for 17 h and protected from light from this point on. After 2 h of release from the second block, cells were irradiated with 2 Gy and processed for immunofluorescence 4 h post-irradiation. Unless otherwise stated, all immunofluorescence dilutions were prepared in PBS and incubations performed at room temperature with intervening washes in PBS. Cell fixation was carried out by incubation with 4% paraformaldehyde for 10 min followed by 100% ice-cold methanol for 5 min at −20°C. This was succeeded by permeabilization in 0.2% Triton X-100 for 5 min and a quenching step using 0.1% sodium borohydride for 5 min. After blocking for 1 h in a solution containing 10% goat serum and 1% BSA, cells were incubated for 1 h with primary antibodies anti-RAD51 (1 :7000, B-bridge International, #70–001) and anti-cyclin A (1:400, BD Biosciences, #611268) diluted in 1% BSA. Secondary antibodies Alexa Fluor 568 goat anti-rabbit (Invitrogen, #A-11011) and Alexa Fluor 647 goat anti-mouse (Invitrogen, #A-21235) were diluted 1:1000 in 1% BSA and applied for 1 h. Nuclei were stained for 10 min with 1 μg/ml 4,6-diamidino-2-phenylindole (DAPI) prior to mounting onto slides with 90% glycerol containing 1 mg/ml paraphenylenediamine anti-fade reagent. Z-stack images were acquired on a Leica CTR 6000 microscope using a 63× oil immersion objective, then deconvolved and analyzed for RAD51 foci formation with Volocity software v6.0.1 (Perkin-Elmer Improvision). The number of RAD51 foci per cyclin A-positive cells expressing the indicated YFP-PALB2 constructs was scored using automatic spot counting by Volocity software and validated manually. Data from three independent trials (total *n* = 225 cells per condition) were analyzed for outliers using the ROUT method (*Q* = 1.0%) in GraphPad Prism v6.0 and the remaining were reported in a scatter dot plot. Intensity values, also provided by Volocity, of 500 RAD51 foci from a representative trial were normalized to the WT mean and reported in a scatter dot plot. Horizontal lines on the plots designate the mean values.

### CRISPR-LMNA HDR assay

U2OS were seeded in 6-well plates at 200 000 cells per well. Knockdown of PALB2 was performed 6–8 h later with 50 nM siRNA using Lipofectamine RNAiMAX (Invitrogen). Twenty-four hours post-transfection, 1.5 × 10^6^ cells were pelleted for each condition and resuspended in 100 μL complete nucleofector solution (SE Cell Line 4D-Nucleofector™ X Kit, Lonza) to which 1μg of pCR2.1-mRuby2LMNAdonor, 1 μg of pX330-LMNAgRNA2, 1 μg of the peYFP-C1 empty vector or the indicated siRNA-resistant YFP-PALB2 construct, and 150 ρmol siRNA was added. Once transferred to a 100 ul Lonza certified cuvette, cells were transfected using the 4D-Nucleofector X-unit, program CM-104, resuspended in culture media and split into 2 60-mm dishes. One dish was harvested 24 h later for protein expression analysis as described above while cells from the other were trypsinised after 48 h for plating onto glass coverslips. Coverslips were fixed with 4% paraformaldehyde and cells analyzed for expression of mRuby2-LMNA (indicative of successful HR) by fluorescence microscopy (63×) a total of 72 h post-nucleofection. Data are represented as mean relative percentages ± SD of mRuby2-positive cells over the YFP-positive population from 3 independent experiments (total *n* >300 YFP-positive cells per condition).

### Regression and statistical analysis

Scatterplots and linear regression were created to examine the correlation between functional data, with squared correlation coefficients (*R*^2^) used to define the strength of the relationships (GraphPad Prism v6.0). For the mammalian two-hybrid assay and the other *in cellulo* experiments, statistical analysis was performed using GraphPad Prism v6.0 and significance was determined applying one-way ANOVA followed by Dunnett's post *hoc* or, when applicable, Kruskal–Wallis test with Dunn's multiple comparison post-test, as specified in each figure legend. All experiments were replicated in at least three independent experiments. (*) *P* < 0.05; (**) *P* < 0.01; (***) *P* < 0.001 and (****) *P* < 0.0001.

## RESULTS

### A systematic approach to identify variants with deleterious effects on PALB2 functions

In order to gain a better understanding regarding the pathogenicity of variants of uncertain significance (VUS) in the *PALB2* tumor suppressor gene, a global functional analysis of variants was undertaken. To this end, 44 missense variants found in breast cancer patients were identified in the ClinVar database (https://www.ncbi.nlm.nih.gov/clinvar) and/or selected by literature curation based on their frequency of description or amino acid substitution position in the protein (Supplemental Table S1). The list was comprised mostly of variants listed as either conflicting or of unknown significance in the Clinvar database, as well as three variants (p.P864S, p.V932M, p.G998E) with a benign/likely benign classification. As a negative control, we included the p.L35P variant, for which pathogenicity has been recently demonstrated (41). When submitted to *in silico* analysis using classical prediction tools (PolyPhen-2 (48) and Align GVGD (49)), 75% of the variants presented at least one score that predicted a potentially damaging effect on the protein function (Supplemental Table S1), which reinforced the need for functional characterization of all 44 *PALB2* missense variants. Three additional and more recent *in silico* prediction algorithms, VEST 3.0 (50) and the meta-predictors M-CAP (51) and REVEL (52), were used for further correlation with the functional assays (Supplemental Table S2).

**Figure 1.**
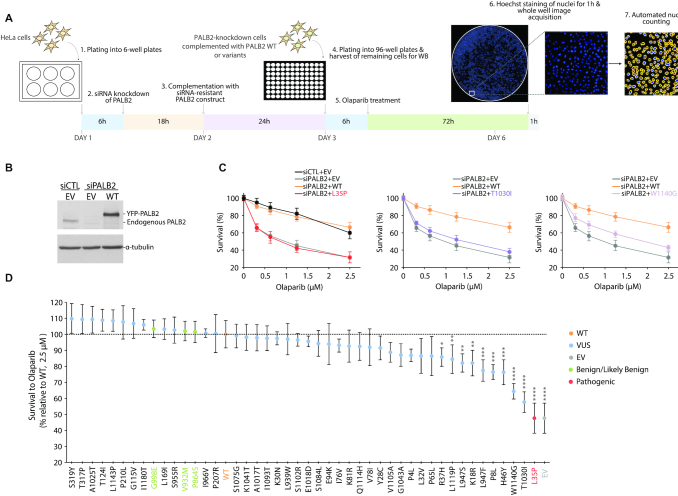
Sensitivity of *PALB2* variants to PARP inhibitor olaparib. PALB2-knockdown HeLa cells, previously transfected with the empty vector (EV) or a siRNA-resistant PALB2 construct (either wild-type (WT) or variant), were seeded into 96-well plates and exposed to olaparib concentrations ranging from 0 to 2.5 μM. Cell viabilities were obtained 72 h post-treatment by quantification of surviving Hoechst-stained nuclei and represented as percent survival relative to the control (DMSO-treated) condition. (**A**) Schematic representation of the strategy employed for testing olaparib sensitivity. (**B**) Typical levels of PALB2 after knockdown and re-expression in HeLa cells, with tubulin as loading control. (**C**) Survival curves contrasting the abilities of PALB2 WT and the p.L35P, p.T1030I and p.W1140G variants to rescue olaparib resistance in PALB2-knockdown cells. (**D**) Olaparib sensitivity profiles for the complete set of variants at a concentration of 2.5 μM, with the WT condition set at 100%. PALB2 variants are classified in descending order of olaparib sensitivity and survival data are presented as the mean (± SD) from at least 3 independent experiments, each performed in triplicate. Statistical significance was determined by Kruskal–Wallis test with Dunn's multiple comparison post-test. (*) *P*< 0.05; (**) *P*< 0.01; (***) *P*< 0.001 and (****) *P*< 0.0001.

To gain further functionality insights, we applied the complete set of *PALB2* missense variants to a PARP inhibitor sensitivity assay in human cells, taking advantage of the synthetic lethal relationship between loss of PALB2 function and PARP inhibition. In this assay, HeLa cells depleted in PALB2 by siRNA-mediated RNA interference were complemented with exogenous expression of siRNA-resistant YFP-PALB2 wild-type (WT) or variants and assayed for olaparib sensitivity (Figure 1A-D). Consistent with previous findings, HR-deficient PALB2-knockdown cells showed marked vulnerability to olaparib due to synthetic lethality and complementation with PALB2 WT restored sensitivity to endogenous level (Figure 1C). Failure to do so by the PALB2 p.L35P pathogenic missense variant corroborated previous observations (41) (Figure 1C). Although the vast majority of variants were almost equivalent to the WT condition, in terms of ability to rescue the viability of PALB2-knockdown cells, the p.T1030I and p.W1140G variants stood out with the highest olaparib sensitivity (Figure 1C), with a survival percentage of 58% and 64% relative to the WT at a dose of 2.5 μM, respectively (Figure 1D). Under these conditions, the relative survival for the empty vector (EV) or p.L35P control was just below 50%. Moderate but statistically significant PARPi sensitivity was observed for seven other variants, i.e. p.P8L, p.K18R, p.R37H, p.H46Y, p.L947F, p.L947S and p.L1119P, providing evidence in favor of a possible functional defect (Figure 1D and Supplemental Figure S1A). Survival curves for variants showing near WT-level of resistance are depicted in Supplemental Figure S2. Variants designated benign/likely benign in ClinVar, p.P864S, p.V932M and p.G998E, fell into this resistant category. For further validation, variants with the strongest phenotype were tested in the human U2OS osteosarcoma cell line. Sensitivities similar to those obtained in HeLa cells were observed. (Supplemental Figure S1B).

In terms of expression in HeLa cells, all variants tested gave rise to a protein product, as detected by immunoblotting 24 h post-transfection (Supplemental Figure S3). Although certain variants were weakly expressed compared to the WT, the expression level appeared a poor predictor of olaparib sensitivity as many variants, including p.E94K, p.P864S, p.I966V, p.A1017T, p.L1143P and p.I1180T, show low expression but WT-level of resistance (Supplemental Figure S2 and S3).

### Functional validation of *PALB2* variants by a two-hybrid assay

**Figure 2.**
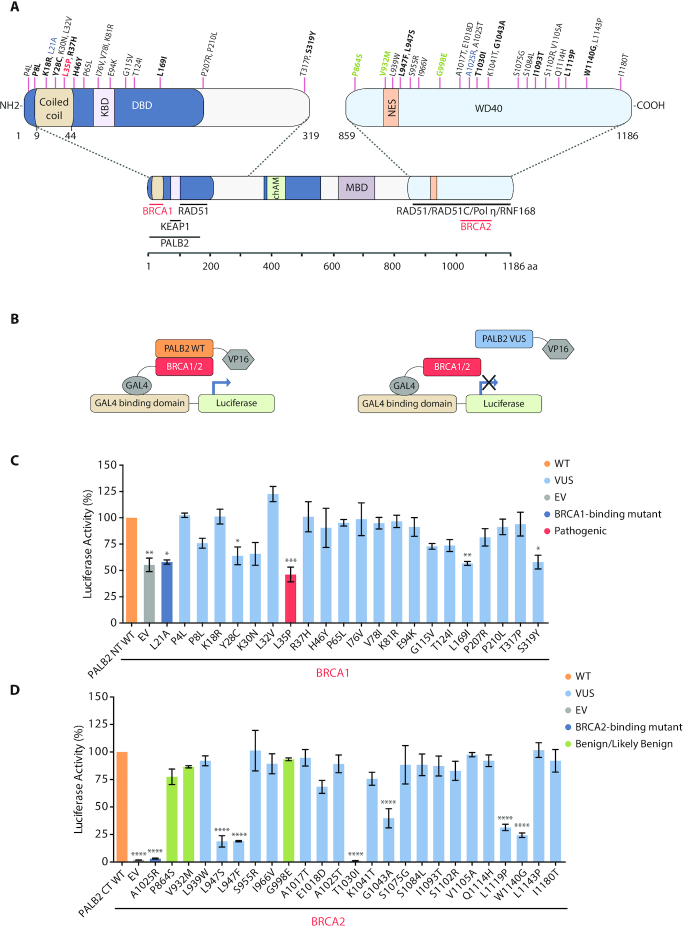
Impact of *PALB2* variants on BRCA1 and BRCA2 interaction by mammalian two-hybrid assay. (**A**) Schematic representation of the PALB2 protein and its domains along with the position of the N- and C-terminal missense variants selected in this study. Negative controls for BRCA1 and BRCA2 binding are in blue and the final prioritized variants in bold, with the p.L35P pathogenic variant highlighted in red and the benign/likely benign variants (as assigned by ClinVar) in green. PALB2 N- (aa 1–319) and C-terminal (aa 859–1186) interactors are also represented. KBD, KEAP1-binding domain; DBD, DNA-binding domain; NES, nuclear export signal; MBD, MRG15-binding domain. (**B**) Mammalian two-hybrid assay representation. (**C**) BRCA1 (aa 1315–1863) and (**D**) BRCA2 (aa 1–60) interaction data; HEK293FT cells were co-transfected with BRCA1/2 and PALB2 N/C-terminal constructs and the reporter assay was performed 24 h post-transfection. Statistical significance was accessed by one-way ANOVA followed by Dunnett's post hoc analysis (mean ± SEM of three independent experiments. (*) *P*< 0.05; (**) *P*< 0.01; (***) *P*< 0.001 and (****) *P*< 0.0001.

Interestingly, the variations associated with the highest sensitivity to olaparib fell either in/near the N-terminal coiled-coil domain or the C-terminal WD40 domain of PALB2 (Figure 2A), while variants outside these regions showed near WT-levels of resistance or slight increases of sensitivity to PARPi that were not statistically significant. The N-terminus of PALB2 interacts with BRCA1 while the WD40 domain has been shown to contain the BRCA2 binding site (6). Based on this, we tested the ability of *PALB2* VUS to interact with BRCA1 or BRCA2 in a mammalian two-hybrid assay (Supplemental Table S3). HEK293FT cells were co-transfected with VP16 AD-PALB2 N-terminal (aa 1–319, WT or variants) and GAL4 DBD-BRCA1 (aa 1315–1863) or VP16 AD-PALB2 C-terminal (aa 859–1186, WT or variants) and GAL4 DBD-BRCA2 (aa 1–60) (Figure 2B). Specifically, 21 variants located in the N-terminus of PALB2 were tested for interaction with BRCA1 and 24 variants in PALB2 C-terminus were analyzed for BRCA2 binding (Figure 2A, C and D). The BRCA1-binding mutant p.L21A and the BRCA2-binding mutant p.A1025R were added in the analysis as negative controls (13,29). As shown in Figure 2C, the interaction between PALB2 WT and BRCA1 increases the luciferase-reporter activity by 2-fold when compared with BRCA1 intrinsic activity alone. In the coiled-coil region, the p.L35P variant showed a reduction in BRCA1 interaction similar to the negative control (p.L21A). The p.Y28C variant also presented impaired BRCA1 binding capacity, corroborating Foo *et al.* previous data (41). Interestingly, two variants (p.L169I and p.S319Y) located outside the coiled-coil domain (BRCA1 minimal interaction region (11–13) also showed reduced activity. Other variants in the N-terminal behaved similarly to the WT control or presented a lesser, statistically nonsignificant reduction.

Regarding BRCA2 interaction analysis, the p.L947F, p.L947S, p.T1030I, p.G1043A, p.L1119P and p.W1140G variants located in the WD40 domain (C-terminal) showed a substantial reduction when compared with WT control activity (Figure 2D). Whereas p.G1043A sustains almost 50% of BRCA2 interaction activity, p.L947F, p.L947S, p.L1119P and p.W1140G exhibit ∼25% of the WT profile. Likewise, the p.T1030I variant, which was previously reported to be an unstable variant (30), also displayed a non-interaction phenotype. The remaining variants in the C-terminal presented a WT-like profile or a moderate reduction in activity, but not statistically significant. Immunoblotting analysis confirmed the ectopic protein production of all 44 PALB2 missense variants (Supplemental Figure S4).

### Prioritization of variants and *in silico* prediction analysis

Based on PARPi sensitivity and PALB2-BRCA1/2 interaction data, we prioritized a smaller set of *PALB2* variants, i.e. p.P8L [c.23C>T], p.K18R [c.53A>G], p.Y28C [c.83A>G] p.R37H [c.110G>A], p.H46Y [c.136C>T], p.L169I [c.505C>A], p.S319Y [c.956C>A], p.L947F [c.2841G>T], p.L947S [c.2840T>C], p.T1030I [c.3089C>T], p.G1043A [c.3128G>C], p.L1119P [c.3356T>C] and p.W1140G [c.3418T>C] (Figure 2A), for further functional assessment. All these selected missense variants were also predicted to be damaging by at least one *in silico* algorithm, except for p.P8L, which was scored as benign or neutral but had shown a significant PARPi sensitivity in our assay. Variants p.K18R, p.Y28C, p.R37H, p.H46Y, p.L169I, p.S319Y, p.L947F, p.L947S and p.G1043A received divergent pathogenicity predictions, whereas p.L1119P and p.W1140G were assigned the highest probability of being pathogenic (Supplemental Table S1 and S2). We also selected the p.I1093T [c.3278T>C] VUS, which was predicted as probably damaging *in silico (*by PolyPhen-2 and VEST 3.0) but showed WT-level of olaparib sensitivity and BRCA2 interaction. It served as variant with likely intact HR function, along with benign/likely benign control variants p.P864S [c.2590C>T], p.V932M [c.2794G>A] and p.G998E [c.2993G>A]) (herein after referred to as the B/BL group). The p.L35P variant, whose interaction with BRCA1 is impaired, was included as a control for HR deficiency. The p.T1030I variant, previously known to be unstable and to abrogate PALB2 association with RAD51 and RAD51C, was expected to behave as another control for HR impairment (30).

At the molecular level, some of these variants introduced a considerable change in amino acid size (Supplemental Figure S5). For an overview of the predicted structural impact of the prioritized VUS, we took advantage of the online tool HOPE (http://www.cmbi.umcn.nl/hope/), designed for automatic mutant analysis and which collects data from multiple sources including the protein 3D-structure and the UniProt database of well-annotated protein sequences. In addition to a change in size, some variants were found to induce a change in hydrophobicity or charge. Namely, the p.Y28C and p.H46Y variants introduce a more hydrophobic residue that can cause loss of hydrogen bonds, disturb correct folding and/or loss of interactions. The arginine to histidine substitution seen with the p.R37H variant changes the residue charge from positive to neutral, which can also cause loss of interactions with other molecules or residues. In the case of the p.L35P variant, the replacement of a leucine by a proline is predicted to act as α-helix breaker that severely impacts on the protein structure. Of particular use, HOPE provided images of the 3D structure of PALB2 WD40 domain (Supplemental Figure S5). At position 947, replacing a leucine with a smaller serine residue can cause an empty space in the core of the protein with loss of hydrophobic interactions while a bulky phenylalanine may not to fit in the protein core. The p.G1043A variant causes the loss of the flexibility conferred by the glycine residue, a property important to allow torsion angles and maintain the proper protein local structure. Replacement of isoleucine by threonine at residue 1093 can cause an empty space in the core of the protein with loss of hydrophobic interactions. The p.L1119P is associated to potential loss of external interactions due to the smaller size of the introduced proline compared to leucine. Finally, major structural changes that could significantly impact on PALB2 interaction and function are reported for the p.T1030I and p.W1140G variants. The p.T1030I variant introduces a residue that is bigger and more hydrophobic than the WT, which is susceptible to disrupt the native hydrogen bond formed with the glutamic acid at position 1011. In line with previous reports on p.T1030I stability, HOPE predicts the threonine to isoleucine substitution to cause an overall destabilization of the protein as a result of incorrect folding. For its part, the mutation of a tryptophan into a smaller and more hydrophobic glycine at position 1140 is predicted to create an empty space in the core of the protein, provide unwanted flexibility and disrupt the native hydrogen bond formed with a cysteine at position 1109.

### PALB2 cellular localization and recruitment kinetics

**Figure 3.**
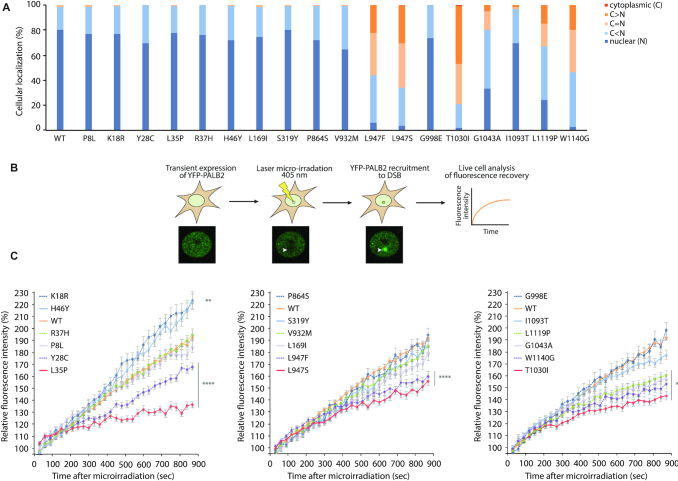
Subcellular localization and recruitment of *PALB2* variants to DNA damage. (**A**) Subcellular localization of YFP-PALB2 missense variants compared to the WT protein (*n* > 120 cells per condition). (**B**) Schematic representation of the laser micro-irradiation experiment used for analyzing the recruitment kinetics of PALB2. (**C**) Quantitative evaluation of recruitment kinetics for YFP-PALB2 WT or missense variants to laser-induced DSBs. Mean curves ± SEM are shown (*n* > 60 cells per condition). Statistics were performed on the last time point (900 s time point) using Kruskal–Wallis test followed by Dunn's multiple comparison post-test. Data from A and C are from at least three independent experiments in HeLa cells. (**) *P*< 0.01 and (****) *P*< 0.0001.

The correlation between the above results and HR functionality for the variants of interest was next explored using complementary functional characterization assays. To begin, the cellular localization of ectopically expressed YFP-PALB2, WT or variants, was assessed in HeLa cells. While p.P8L, p.K18R, p.Y28C, p.L35P, p.R37H, p.H46Y, p.L169I, p.S319Y, p.G1043A, p.I1093T and the B/BL group appeared almost strictly nuclear, as the WT, we observed moderate cytoplasmic accumulation of p.L947F, p.L947S, p.L1119P, p.W1140G and a very substantial one for p.T1030I (Figure 3A). It is likely that this mislocalization of PALB2 occurs as a result of an unmasking of the NES caused by the latter variations, as suggested for other WD40-domain mutants (32).

We next proceeded to monitor the recruitment of YFP-PALB2 to laser-induced DNA damage sites by live-cell imaging (Figure 3B). To do this, we micro-irradiated populations of nuclei expressing YFP-PALB2 and quantified fluorescence accumulation at the damage sites with respect to time. Recruitment kinetics analysis of the N-terminus variants, all strictly nuclear, revealed recruitment defects for variant p.Y28C and p.L35P (Figure 3C). At 15 min post-irradiation (900 s time-point), p.Y28C accumulation at the micro-irradiated site reached 75% of that of WT, which was intermediate compared to the 40% accumulation seen for p.L35P. For the p.P8L, p.R37H, p.L169I and p.S319Y variants, we found no statistical differences in recruitment compared to WT, whereas p.K18R and p.H46Y showed enhanced assembly. In C-terminus, p.T1030I, the variant exhibiting the most striking mislocalization, had also the most severe recruitment defect. Its accumulation had reached only 47% of WT level at 15 min, while that of p.L947F, p.L947S, p.G1043A, p.L1119P and p.W1140G attained between 60–65% approximately. The B/BL group was recruited with kinetics similar to WT. While the recruitments defects seen in PALB2 C-terminus variants could be attributed to nuclear exclusion, defects observed for the nuclear, N-terminus variants p.Y28C and p.L35P might result from a loss of interaction, most likely with BRCA1 or PALB2 itself (41).

### RAD51 foci formation and CRISPR-LMNA HDR assays

**Figure 4.**
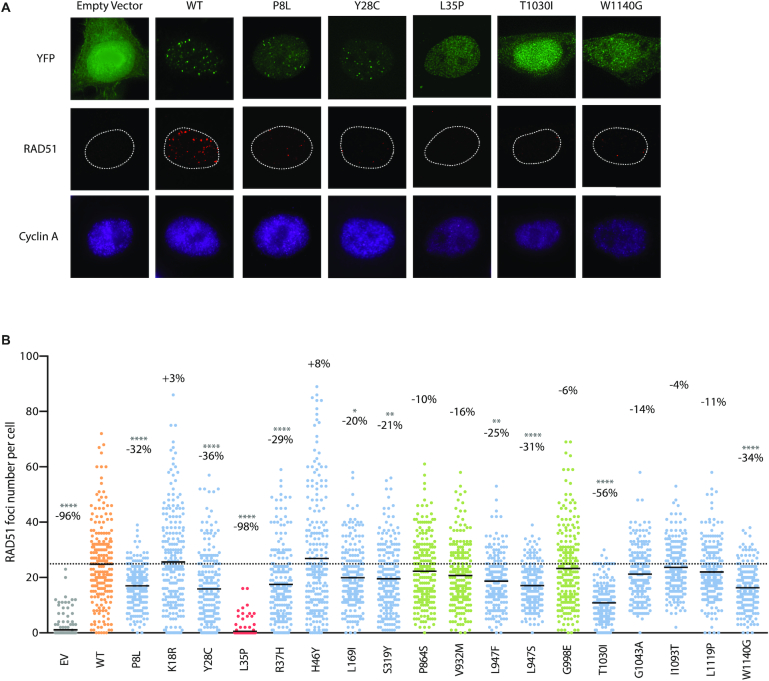
RAD51 foci formation in *PALB2* variants. (**A**) Representative immunofluorescence images of RAD51 foci (red) in PALB2-knockdown HeLa cells complemented with the empty vector or the indicated siRNA-resistant YFP-PALB2 construct (green) and synchronized in S/G2 by double thymidine block, as determined by cyclin A co-staining (purple). Images were acquired from cells fixed 4 h post-ionizing radiation (2 Gy). Variants leading to the strongest phenotypes are shown. (**B**) RAD51 foci quantification from images in (A) using Volocity software. The scatter dot plot shows the number of RAD51 foci in cyclin A-positive cells expressing the indicated YFP construct, with the horizontal lines designating the mean values of three independent trials (*n* = 225 cells per condition). The percentage change relative to the WT mean is also indicated for each variant. VUS are depicted in blue, p.L35P pathogenic variant in red, benign/likely benign variants in green, while wild-type PALB2 and the empty vector are highlighted in orange and grey, respectively. Statistical significance was determined by Kruskal–Wallis test with Dunn's multiple comparison post-test. (*) *P*< 0.05; (**) *P <* 0.01 and (****) *P*< 0.0001.

**Figure 5.**
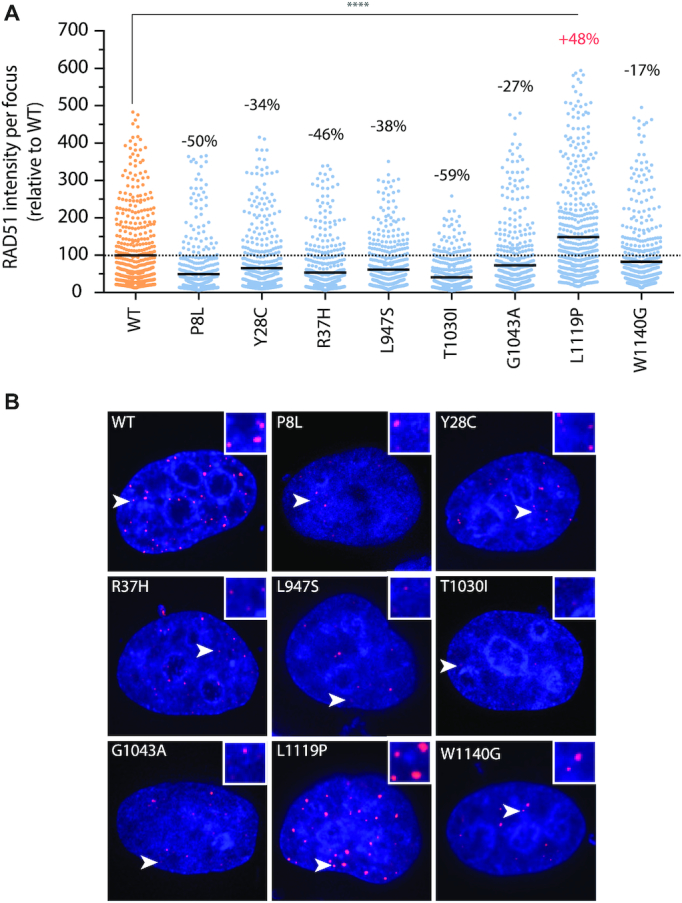
*PALB2* variants showing altered RAD51 foci intensity. (**A**) Distribution of fluorescent intensities of RAD51 foci as determined using Volocity software. The scatter dot plot shows the intensity values (relative to the WT mean) of 500 RAD51 foci from a representative trial, with the horizontal lines designating the mean values. The percentage change relative to the WT mean is also indicated for each variant. VUS are depicted in blue and wild-type PALB2 in orange. Statistics were performed by Kruskal–Wallis test followed by Dunn's multiple comparison post-test. (****) *P*< 0.0001. (**B**) Representative images with enlarged insets of altered RAD51 foci intensity. Merge images of DAPI (blue) and RAD51 (red) staining are shown.

For a more direct readout of HR competency, we pursued our functional analysis using RAD51 foci formation and CRISPR-LMNA (Lamin A/C) HDR (homology-directed repair) assays. As in PARPi sensitivity assays, these were carried out by exogenous expression of siRNA-resistant YFP-PALB2 WT or variant, in a background of endogenous PALB2 depletion by siRNA-mediated RNA interference. RAD51 foci formation was quantified following treatment with 2 Gy of ionizing radiation in S/G2-synchronized HeLa cells (cyclin A-positive cells). As expected, a dramatic decrease of over 95% in the mean number of RAD51 foci was observed for the YFP-empty vector and p.L35P. Variants p.P8L, p.Y28C, p.R37H, p.L947F, p.L947S, p.T1030I and p.W1140G also showed considerable decreases ranging from 56% for p.T1030I to 25% for p.L947F, while the other elicited modest to no decrease (Figure 4). For certain variants, the RAD51 foci that remained were also weaker in intensity (Figure 5). Unlike the others, however, p.L1119P exhibited larger and brighter RAD51 foci compared to the WT, pointing to a possible defect in RAD51 foci disassembly.

**Figure 6.**
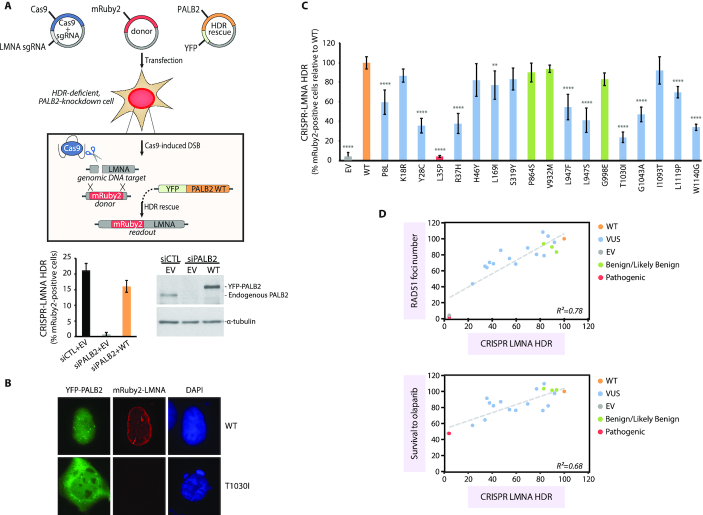
HDR activity of *PALB2* variants using the CRISPR-LMNA system. (**A**) Schematic representation of the system. The CRISPR-LMNA system measures the HDR-dependent insertion of mRuby2 into a Cas9-mediated DSB in the *LMNA* gene, resulting in cellular expression of mRuby2-tagged lamin A/C (LMNA) that serves as readout for HDR activity. Also shown is the typical level of HDR activity of PALB2 after siRNA knockdown and complementation in U2OS cells, with accompanying immunoblots. Data in the bar graph represents mean percentages (± SD) of mRuby2-positive cells among the cell population expressing the YFP-empty vector or siRNA-resistant PALB2 WT, as determined by fluorescence microscopy 72 h post-nucleofection of the CRISPR-LMNA system. (**B**) Representative fluorescence microscopy image of a cell expressing mRuby2-LMNA after successful homology directed repair, as seen in PALB2-depleted cells complemented with siRNA-resistant YFP-PALB2 WT *(top)*. Cell negative for mRuby2-LMNA expression as typically found after complementation with the p.T1030I variant *(bottom)*. (**C**) Quantification of HDR activity after complementation of PALB2-knockdown U2OS cells with empty vector or the indicated siRNA-resistant PALB2 construct. Data represents mean relative percentages (± SD) of mRuby2-positive cells among the YFP-positive population from 3 independent experiments (*n* > 300 YFP-positive cells per condition) relative to the WT condition. VUS are depicted in blue, p.L35P pathogenic variant in red, benign/likely benign variants in green, while PALB2 wild-type and the empty vector are highlighted in orange and grey, respectively. Statistical significance was accessed by one-way ANOVA followed by Dunnett's post hoc analysis. (**) *P*< 0.01 and (****) *P*< 0.0001. (**D**) Scatter graphs with regression lines (in grey) correlating HDR activity with the mean number of RAD51 foci per cell or cell survival to olaparib. Values are expressed in percentage relative to WT (set to 100%). *R*^2^ values are shown.

Lastly, HR functionality was investigated in U2OS cells, using a modified version of the previously described CRISPR-LMNA HDR assay (46) that generates a red fluorescent mRuby2-Lamin A/C fusion upon successful HR (Figure 6A and B). Supplemental Figure S6 presents typical knockdown and complementation in U2OS cells. Under these settings, knockdown of PALB2 decreased the absolute percentage of HDR from 21% to 0.7% and complementation with PALB2 WT restored the level to 16%. To best appreciate the impact of the variants, results were reported relative to the WT condition. Hence, we observed the YFP-empty vector and p.L35P negative controls reduced HR activity ∼95% relative to the WT condition (Figure 6C). Again, p.T1030I and p.W1140G showed substantial incapacity to promote HR, retaining only 23.6% and 34.0% of HR activity respectively, followed by p.Y28C and p.R37H whose activities were under 40% as well. Other variants showed more intermediate phenotypes, ranging from 41.0% to 77.0% of HR activity, while p.I1093T and the B/LB group were HR proficient. Consistent with RAD51 foci being a marker of functional homologous recombination, we found a robust correlation between HDR activity and RAD51 foci formation (*R*^2^ = 0.78), and this no matter the domain involved (Figure 6D and Supplemental Figure S7). These data also underscored the importance of the coiled-coil and the WD40 domains for PALB2 function in DSB repair. Regarding the relationship between HDR functionality and olaparib resistance (*R*^2^ = 0.68), a much higher *R*-squared value (*R*^2^ = 0.85) was obtained when variants located in the region of the WD40 domain were analyzed separately.

## DISCUSSION

In absence of a universal functional assay for the classification of *PALB2* variants of uncertain significance, we have investigated the biological consequences of 44 missense variants using a combination of techniques, including PARP inhibitor sensitivity, *in silico* prediction tools, protein-interaction and HDR assays. The collection of methods described here provides a framework for exon-wide *PALB2* deleteriousness assignment.

Several considerations were taken while designing the choice of functional assays. For the PARPi sensitivity assays, olaparib (Lynparza) was selected knowing that the FDA had approved the compound for the treatment of patients with germline *BRCA*-mutated, HER2-negative metastatic breast cancer who have previously received chemotherapy. Mouse ES-cell-based functional assays have been frequently used to evaluate VUS. However, human and mouse PALB2 are only 58% identical at the amino acid level. Instead, we relied on an homologous complementation system where human PALB2 was expressed in human cancer cell lines depleted for PALB2. We have also used a CRISPR-LMNA HDR assay that is very sensitive to monitor HR at the *LMNA* locus, as HR rates detected with this system routinely attain 15–25% (53). This provides a strong signal to noise ratio that is needed when comparing several variants, and especially those with intermediate phenotypes.

Our study uncovered two hotspots for *PALB2* missense variants leading to defects in homologous recombination. At the N-terminus of PALB2, our analysis identified p.P8L, p.Y28C, and p.R37H as missense variations with compromised HR activity. These variants are located near/within the coiled-coil domain of PALB2, which is involved in the heterodimerization of the protein with BRCA1. In the case of p.Y28C, although not correlated with PARPi sensitivity in agreement with Foo *et al.* (41), the partial loss of interaction with BRCA1 observed in our two-hybrid analysis could provide an explanation for the HR deficit as well as the intermediate reduction in PALB2 recruitment to DNA damage sites. A variation in the coiled-coil motif may also target PALB2 self-oligomerization activity. Recent work by Song *et al.* demonstrated that the PALB2 homodimer is mediated by an anti-parallel coiled-coil structure and identified L17, L21, L24, Y28, T31 and L35 as key hydrophobic residues at the dimer interface (54). Mutation of L24 significantly reduces the homodimer stability and impacts on PALB2 DNA repair activity. Since the p.P8L and p.R37H variants show weak to null impact on BRCA1 binding, normal recruitment to DNA breaks, and do not target key interface residues of the PALB2 coiled-coil homodimer interaction, a downstream mechanism could be responsible for their effect on HR. Close to the coiled-coil motif of PALB2 is the RAD51-binding site (14,15). It is possible that the PALB2-RAD51 interaction might be compromised with these missense mutations. Functional aspects of the N-terminus and coiled-coil motif need to be further explored. For instance, variants p.K18R and p.H46Y are of interest as they were recruited more efficiently to laser-induced DSBs than wild-type PALB2 and HR proficient, but yet showed increased sensitivity to olaparib. Hence, the correlation between the HR competency and olaparib sensitivity could not be applied to all variants analyzed suggesting other functions of PALB2 leading to PARPi sensitivity.

In C-terminal, PALB2 missense variants p.L947F, p.L947S, p.T1030I and p.W1140G were found to interfere with HR and every activity tested. These all fall in the WD40 domain. In a previous study, we reported that a truncating mutation in the WD40 domain of PALB2, known as p.W1038X, causes a defective nuclear localization of the protein. More specifically, we have uncovered that the PALB2 WD40 domain hides a Nuclear Export Sequence (NES) that can be exposed when a truncation within this domain occurs (32), leading to the cytoplasmic accumulation of PALB2. Interestingly, this model is also recapitulated in some of the *PALB2* variants analyzed in the current study, although they bear a simple point mutation. Indeed, a proportion of PALB2 p.L947F, p.L947S, p.T1030I and p.W1140G accumulated into the cytoplasm, a phenotype accompanied by a significant reduction in homologous recombination activity. We also observed that the expression levels of these WD40 variants were reduced compared to wild-type PALB2, perhaps indicating that they might be unstable and undergo some degradation in the cytoplasm. Some other WD40 variants, such as p.L1143P and p.I1180T, showed also lower expression, but were resistant to olaparib, suggesting that mutations in the WD40 do not automatically lead to protein instability. Our data suggest that targeting PALB2 nuclear export with inhibitors of the nuclear export receptor CRM1, such as Selinexor (KPT-330), could be of interest to drive the p.L947F, p.L947S, p.T1030I or p.W1140G variants in the nucleus as a potential therapeutic avenue for patients harboring these missense mutations. Selinexor is an orally bioavailable selective inhibitor of nuclear export that is currently in Phase I and II clinical trials for advanced cancers. Furthermore, it has shown anti-tumor efficacy in preclinical models of triple-negative breast cancer (55).

Importantly, the WD40 domain acts as a critical protein interaction scaffold for PALB2 activity in DSB repair. It is the site of interaction for several DNA repair factors, including, BRCA2 (29), RAD51 and RAD51AP1 (14,15), RAD51C (30), Pol η (21) and RNF168 (31). Hence, missense mutations in the WD40 might impair interactions with these factors. For instance, the p.G1043A variant displayed a recombination defect despite normal nuclear localization. This variant maps in the BRCA2-interaction region and therefore might affect BRCA2 loading on chromatin, as supported by our two-hybrid analysis. Interestingly, p.G1043A and p.L1119P have similar defects in binding BRCA2 through two-hybrid assay, similar nuclear and cytoplasmic distributions, and a similar decrease in the number of RAD51 foci per cell. However, p.L1119P is slightly more efficient at HR repair than p.G1043A but shows less resistance towards PARPi. Variants p.L1119P and p.G1043A might have different mechanistic defects even physically present in the same cluster. In particular, p.L1119P expression leads to larger RAD51 foci perhaps indicating a defect in RAD51 disassembly.

**Figure 7.**
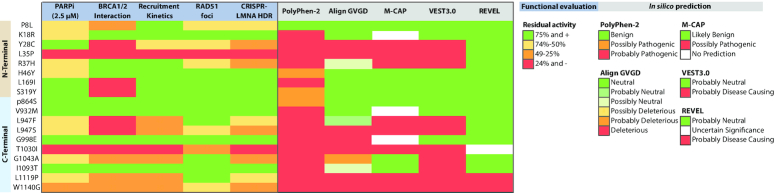
Profile of functionality of prioritized *PALB2* variants. Heat map summarizing the percentage of residual activity for each *PALB2* VUS and its predicted pathogenicity profile. In order to facilitate interpretation of the functional data, values were rescaled to a 0–100% scale for each assay, with the mean value for known pathogenic controls set to 0% and the mean value for non-pathogenic controls to 100%. Where applicable, the pathogenic controls considered for mean value calculations were the empty vector and the p.L35P variant while the wild-type and the benign/likely benign variants, p.P864S, p.V932M and p.G998E, were used as non-pathogenic controls. For BRCA1 and BRCA2 interaction, p.L21A and p.A1025R were also taken into account as pathogenic controls.

Of note, some discordance could be observed between *in silico* predictions and our functional analysis. Out of the 44 studied variants, 33 were predicted to have a potentially damaging effect on *PALB2* by at least one of the five *in silico* tools used (Supplemental Table S1 and S2), while our systematic approach highlighted about a dozen with weakly to strongly compromised functions. This discrepancy was mainly attributable to PolyPhen-2, which seemingly, largely overestimated the number of variants with functional impact (Supplemental Figure S1), suggesting this tool may lead to false-positive predictions and is perhaps not indicated to guide the clinical decision alone. Conversely, the more restrictive algorithm REVEL classified all but two variants (p.L1119P and p.W1140G) as neutral, implying a propensity for false-negative predictions (Supplemental Table S2). In line with this, all tools appeared as poor predictors of olaparib responsiveness, as judged by *R*-squared values (≤0.33) from the regression analysis (Supplemental Figure S8). To facilitate interpretation of the prioritized variants, functional data for each assay were normalized according to mean values from the known benign and pathogenic controls included in the study and variants were categorized based on their percentage of residual activity (Figure 7). According to the profile of functionality obtained, the highest discrepancies with *in silico* predictions were observed with p.P8L. This variant was shown to be functionally compromised in all except the PALB2 recruitment assay but was classified as benign/neutral by all five predictive tools. In addition, p.R37H and p.L947F exhibited compromised function for at least three out of five assays but were scored as neutral by Align GVGD and REVEL. On the contrary, the p.I1093T variant, which conserved intact function according to all assays, was qualified as probably pathogenic/disease-causing by Polyphen-2 and VEST 3.0. Nevertheless, Align GVGD, M-CAP and VEST 3.0 appeared the most consistent with our findings (Figure 7) and thus more reliable for prediction of functional deleteriousness of *PALB2* VUS. In terms of relationships among functional assays, appreciable correlations were observed, the most stringent one being between HDR activity and RAD51 foci-forming ability (*R*^2^ = 0.78 for all prioritized variants, 0.89 for the coiled-coil region, and 0.72 for the WD40 domain). The data also shows a positive correlation between the mean number of RAD51 foci per cell and resistance to olaparib (*R*^2^ = 0.57), with a much higher R-squared value (*R*^2^ = 0.78) obtained when variants located in the region of the WD40 domain are analyzed separately. Of particular relevance is the robust correlation seen between PARPi responsiveness and HDR status for variants in the WD40 domain (*R*^2^ = 0.85), which suggests that PARPi sensitivity can predict functional deleteriousness in the WD40 domain and vice versa. These correlations are recapitulated in the correlation graphs (Supplementary Figure S7).

Our results provide robust evidence for the role of several variants on the cellular function of PALB2. It should be noted however, that the functional assays used in this study have not been validated with regard to the pathogenicity of missense variants in *PALB2* and therefore caution is warranted when interpreting the clinical significance of these variants of unknown significance. Also, most variants we analyzed showed partially compromised PALB2 function leading to intermediate phenotypes and it is not known at this stage whether or not these defects translate into increased cancer risk and therapeutic response. In contrast to BRCA1 and BRCA2, for which functional assays calibrated to breast cancer risk in terms of specificity and sensitivity are available, no such assays currently exist for the classification of *PALB2* variants, mainly due to the lack of family-based data of truly pathogenic missense variants. Once more data is available, such as segregation of the variant with the disease in the family, co-occurrence of the variant with known pathogenic mutations and robust population-based case-control analysis, the use of functional assays in combination with these other data sources will then greatly help expert committees in establishing the clinical relevance of *PALB2* VUS.

Furthermore, the large amount of functional data presented here can now be used in combination with ongoing initiatives (Boonen, R.A.C.M., *et al.* Functional analysis of genetic variants in the high-risk breast cancer susceptibility gene *PALB2* (in press); Wiltshire, T., *et al.* Functional characterization of 84 *PALB2* variants of uncertain significance (in press)) and computational predictive tools to aid determining more accurately the deleteriousness for *PALB2* missense variants. For instance, VarCall was designed as a computational tool that incorporated functional data from the C-terminal domain of BRCA1 to determine the likelihood of pathogenicity for over 300 missense variants (56,57). A similar predictive computational tool could now be generated. Not only would this be a major step in predicting pathogenicity, but also it would help establishing a correlation between PARPi treatment and HR in patients. Recently, RAD51 was shown to be a good marker to predict PARPi sensitivity (47). Our results suggest that PALB2 mediated-HR could be also a good predictive marker for PARPi response. As there are more than 1100 missense mutations in PALB2 reported in ClinVar (https://www.ncbi.nlm.nih.gov/clinvar), our attention will now focus on using the functional analyses presented here with saturation mutagenesis analyses, as described recently (58,59), to assess the global impact of *PALB2* missense mutations.

## Supplementary Material

gkz780_Supplemental_Files
