# Supplementary material for: A global functional analysis of missense mutations reveals two major hotspots in the PALB2 tumor suppressor
Source: Nucleic Acids Res. 2019 Oct 5;47(20):10662–77. doi: 10.1093/nar/gkz780 (PMC6847799; doi:10.1093/nar/gkz780)
Supplement: gkz780_Supplemental_Files [file gkz780_supplemental_files.zip › Supplemental_information_Rodrigue_et_al_.pdf]

A global functional analysis of missense mutations reveals two major hotspots in the *PALB2* tumor suppressor

Amélie Rodrigue[1,2], Guillaume Margaillan[3], Thiago Torres Gomes[4,5], Yan Coulombe[1,2], Gemma Montalban[1,2,3], Simone da Costa e Silva Carvalho[1,4,6], Larissa Milano de Souza[1,2], Mandy Ducy[1,2,3], Giuliana De-Gregoriis[4,5], Graham Dellaire[7], Wilson Araújo da Silva Junior [6], Alvaro Monteiro[8], Marcelo Carvalho[4,5,9], Jacques Simard[3,9], and Jean-Yves Masson[1,2,9].

Supplemental Figures

Supplemental Figure S1. Survival curves for *PALB2* variants showing sensitivity to PARP inhibitor olaparib (red) in HeLa cells (A) and validation in U2OS cells (B). Curves for the wild-type and empty vector are shown in orange and grey, respectively.

Supplemental Figure S2. Survival curves for *PALB2* variants showing resistance to PARP inhibitor olaparib (green) similar to wild-type in HeLa cells. The wild-type and empty vector curves are shown in orange and grey, respectively.

Supplemental Figure S3. Expression levels of *PALB2* variants in HeLa cells. Immunoblotting analysis of *PALB2* missense variants in siPALB2-depleted HeLa cells, 24 h post-complementation with the indicated siRNA-resistant YFP-PALB2 constructs, using PALB2 antiserum and monoclonal anti- α-tubulin antibody as loading control.

Supplemental Figure S4. Expression levels of *PALB2* variants in the mammalian two-hybrid analysis. HEK293FT cells transfected with the indicated constructs were analyzed for protein expression by immunoblotting, 24 h post-transfection using anti-VP16 and anti-GAL4 antibodies.

Supplemental Figure S5. Structural impact of the prioritized variants using HOPE analysis.
A) Schematic structures of the mutated and corresponding WT residues for each variants in the coiled-coil region, with their common backbone (red) and unique side chain (black). B) Close-up images of the WD40 domain obtained from HOPE analysis showing the structural impact of the variants studied in this region. The yellow arrow points out the side chain differences caused by the amino acid change. The protein is in grey, the wild-type and variant side chains are in green and red, respectively. In the bottom right corner is the schematic structure of each residue, with their common backbone (red) and unique side chain (black).

Supplemental Figure S6. Expression levels of prioritized *PALB2* variants in U2OS cells. Immunoblotting analysis of *PALB2* missense variants in siPALB2-depleted U2OS cells, 24 h post-complementation with the indicated siRNA-resistant YFP-PALB2 constructs, using PALB2 antiserum and monoclonal anti- α-tubulin antibody as loading control.

Supplemental Figure S7. Correlations between functional assays. Scatter graphs depicting the correlations between the outcomes of functional assays for all the prioritized variants or categorized by domain. Values are expressed in percentage relative to WT (set to 100%). Regression lines (in grey) and R^2 values are shown.

Supplemental Figure S8. Correlations between *in silico* predictions and response to olaparib. Scatter graphs showing the correlations between *in silico* predictions (by M-CAP, VEST 3.0 and REVEL) and survival to olaparib for all 44 *PALB2* VUS. Survival data are expressed in percentage relative to WT (set to 100%). Regression lines (in grey) and R^2 values are shown.
