# Supplementary material for: A global functional analysis of missense mutations reveals two major hotspots in the PALB2 tumor suppressor
Source: Nucleic Acids Res. 2019 Oct 5;47(20):10662–77. doi: 10.1093/nar/gkz780 (PMC6847799; doi:10.1093/nar/gkz780)
Supplement: gkz780_Supplemental_Files [file gkz780_supplemental_files.zip › Table S1. Functionality screening of PALB2 missense VUS.docx]

**Table S1. Functionality screening of *PALB2* missense VUS**

| ***PALB2* region** | **Mutation** | |  | ***In silico* pathogenicity prediction** | | **ClinVar designation** | **References** |
| --- | --- | --- | --- | --- | --- | --- | --- |
|  | DNA change | Protein Change |  | PolyPhen-2 | Align GVGD ^1^ |  |  |
| N-terminal | c.11C>T | p.P4L |  | Benign (0.090) | Neutral (C0) | Conflicting interpretations of pathogenicity | [1-5] |
| N-terminal | c.23C>T | p.P8L |  | Benign (0.008) | Neutral (C0) | Conflicting interpretations of pathogenicity | [2, 6, 7] |
| N-terminal | c.53A>G | p.K18R |  | Probably Damaging (1.000) | Neutral (C0) | Conflicting interpretations of pathogenicity | [2, 3, 7-10] |
| N-terminal | c.83A>G | p.Y28C |  | Probably Damaging (1.000) | Deleterious (C65) | Uncertain significance | [11] |
| N-terminal | c.90G>T | p.K30N |  | Probably Damaging (0.999) | Neutral (C0) | Uncertain significance | [12] |
| N-terminal | c.94C>G | p.L32V |  | Probably Damaging (1.000) | Neutral (C0) | Conflicting interpretations of pathogenicity | [4, 12] |
| N-terminal | c.104T>C | p.L35P |  | Probably Damaging (1.000) | Deleterious (C65) | ─ | [13] |
| N-terminal | c.110G>A | p.R37H |  | Probably Damaging  (0.968) | Possibly Neutral (C25) | Uncertain significance | [14] |
| N-terminal | c.136C>T | p.H46Y |  | Possibly Damaging  (0.838) | Neutral (C0) | ─ | [15] |
| N-terminal | c.194C>T | p.P65L |  | Benign (0.007) | Neutral (C0) | Uncertain significance | [2-4, 16] |
| N-terminal | c.226A>G | p.I76V |  | Benign (0.000) | Neutral (C0) | Conflicting interpretations of pathogenicity | [2, 17] |
| N-terminal | c.232G>A | p.V78I |  | Benign (0.009) | Neutral (C0) | Conflicting interpretations of pathogenicity | [2-4, 14, 16] |
| N-terminal | c.242A>G | p.K81R |  | Benign (0.037) | Neutral (C0) | Uncertain significance | ─ |
| N-terminal | c.280G>A | p.E94K |  | Possibly Damaging (0.732) | Neutral (C0) | ─ | [1] |
| N-terminal | c.344G>T | p.G115V |  | Benign (0.002) | Neutral (C0) | Conflicting interpretations of pathogenicity | [3-5, 17] |
| N-terminal | c.371C>T | p.T124I |  | Benign (0.001) | Neutral (C0) | Conflicting interpretations of pathogenicity | [1] |
| N-terminal | c.505C>A | p.L169I |  | Probably Damaging (0.972) | Neutral (C0) | Uncertain significance | [18] |
| N-terminal | c.620C>G | p.P207R |  | Possibly Damaging (0.879) | Neutral (C0) | Uncertain significance | [19] |
| N-terminal | c.629C>T | p.P210L |  | Benign (0.021) | Neutral (C0) | Conflicting interpretations of pathogenicity | [1, 2, 4, 7, 20, 21] |
| N-terminal | c.949A>C | p.T317P |  | Possibly Damaging (0.630) | Possibly Deleterious (C35) | Uncertain significance | ─ |
| N-terminal | c.956C>A | p.S319Y |  | Possibly Damaging (0.589) | Neutral (C0) | Uncertain significance | [12] |
| C-terminal | c.2590C>T | p.P864S |  | Possibly Damaging (0.578) | Neutral (C0) | Benign/Likely benign | [1-5, 10, 12, 14-16, 20-33] |
| C-terminal | c.2794G>A | p.V932M |  | Probably Damaging  (1.000) | Neutral (C0) | Benign/Likely benign | [1-5, 8, 10, 11, 14-16, 20, 21, 23-29, 32-37] |
| C-terminal | c.2816T>G | p.L939W |  | Probably Damaging  (1.000) | Probably Deleterious (C55) | Conflicting interpretations of pathogenicity | [1-5, 8-10, 14-16, 20, 21, 23-29, 33, 38-40] |
| C-terminal | c.2840T>C | p.L947S |  | Probably Damaging (1.000) | Deleterious (C65) | Uncertain significance | [1, 41] |
| C-terminal | c.2841G>T | p.L947F |  | Probably Damaging (1.000) | Probably Neutral (C15) | ─ | [1] |
| C-terminal | c.2865T>A | p.S955R |  | Benign (0.051) | Neutral (C0) | Uncertain significance | [19] |
| C-terminal | c.2896A>G | p.I966V |  | Probably Damaging (0.977) | Neutral (C0) | Uncertain significance | [15] |
| C-terminal | c.2993G>A | p.G998E |  | Probably Damaging (1.000) | Deleterious (C65) | Benign/Likely benign | [1, 2, 4, 5, 8, 10, 11, 14-17, 20-23, 25, 26, 28-32, 36-39, 42-46] |
| C-terminal | c.3049G>A | p.A1017T |  | Probably Damaging (1.000) | Neutral (C0) | ─ | [47] |
| C-terminal | c.3054G>C | p.E1018D |  | Probably Damaging (0.998) | Neutral (C0) | Conflicting interpretations of pathogenicity | [4, 9, 33, 47-52] |
| C-terminal | c.3073G>A | p.A1025T |  | Possibly Damaging (0.944) | Probably Deleterious (C55) | Uncertain significance | [15] |
| C-terminal | c.3089C>T | p.T1030I |  | Probably Damaging (1.000) | Deleterious (C65) | Uncertain significance | [15] |
| C-terminal | c.3122A>C | p.K1041T |  | Possibly Damaging (0.661) | Neutral (C0) | Uncertain significance | [53] |
| C-terminal | c.3128G>C | p.G1043A |  | Probably Damaging (1.000) | Probably Deleterious (C55) | Uncertain significance | [2-4, 15, 27, 40] |
| C-terminal | c.3223A>G | p.S1075G |  | Probably Damaging (0.986) | Neutral (C0) | ─ | [15] |
| C-terminal | c.3251C>T | p.S1084L |  | Benign (0.008) | Neutral (C0) | Uncertain significance | [1-5] |
| C-terminal | c.3278T>C | p.I1093T |  | Probably Damaging (0.999) | Possibly Neutral (C25) | Uncertain significance | [1, 9, 54] |
| C-terminal | c.3306C>G | p.S1102R |  | Probably Damaging (0.976) | Neutral (C0) | Uncertain significance | [48] |
| C-terminal | c.3314T>C | p.V1105A |  | Probably Damaging (0.999) | Possibly Neutral (C25) | ─ | [15] |
| C-terminal | c.3342G>C | p.Q1114H |  | Possibly Damaging (0.831) | Neutral (C0) | Uncertain significance | [15] |
| C-terminal | c.3356T>C | p.L1119P |  | Probably Damaging (1.000) | Deleterious (C65) | Uncertain significance | [9] |
| C-terminal | c.3418T>G | p.W1140G |  | Probably Damaging  (1.000) | Deleterious (C65) | Uncertain significance | ─ |
| C-terminal | c.3428T>C | p.L1143P |  | Possibly Damaging (0.562) | Neutral (C0) | Uncertain significance | [15] |
| C-terminal | c.3539T>C | p.I1180T |  | Probably Damaging (1.000) | Possibly Neutral (C25) | Uncertain significance | [43] |

^1^ Align GVGD scores range from C0 to C65, with C0 being the less likely and C65 the most likely to interfere with protein function; The scores were classified as Neutral, Probably Neutral, Possibly Neutral, Possibly Deleterious, Probably Deleterious and Deleterious by the authors, according to the predicted scores and to the tool’s terminology. The variants without reference were selected only based on the ClinVar data.

References

1. Rahman, N., et al., *PALB2, which encodes a BRCA2-interacting protein, is a breast cancer susceptibility gene.* Nat Genet, 2007. **39**(2): p. 165-7.

2. Ramus, S.J., et al., *Germline Mutations in the BRIP1, BARD1, PALB2, and NBN Genes in Women With Ovarian Cancer.* J Natl Cancer Inst, 2015. **107**(11).

3. Damiola, F., et al., *Mutation analysis of PALB2 gene in French breast cancer families.* Breast Cancer Res Treat, 2015. **154**(3): p. 463-71.

4. Thompson, E.R., et al., *Prevalence of PALB2 mutations in Australian familial breast cancer cases and controls.* Breast Cancer Res, 2015. **17**: p. 111.

5. Wong-Brown, M.W., et al., *Low prevalence of germline PALB2 mutations in Australian triple-negative breast cancer.* Int J Cancer, 2014. **134**(2): p. 301-5.

6. Cock-Rada, A.M., et al., *A multi-gene panel study in hereditary breast and ovarian cancer in Colombia.* Fam Cancer, 2018. **17**(1): p. 23-30.

7. Ding, Y.C., et al., *Germline mutations in PALB2 in African-American breast cancer cases.* Breast Cancer Res Treat, 2011. **126**(1): p. 227-30.

8. Bogdanova, N., et al., *PALB2 mutations in German and Russian patients with bilateral breast cancer.* Breast Cancer Res Treat, 2011. **126**(2): p. 545-50.

9. Nguyen-Dumont, T., et al., *Mutation screening of PALB2 in clinically ascertained families from the Breast Cancer Family Registry.* Breast Cancer Res Treat, 2015. **149**(2): p. 547-54.

10. Tischkowitz, M.D., et al., *Analysis of the gene coding for the BRCA2-interacting protein PALB2 in familial and sporadic pancreatic cancer.* Gastroenterology, 2009. **137**(3): p. 1183-6.

11. Ding, Y.C., et al., *Mutations in BRCA2 and PALB2 in male breast cancer cases from the United States.* Breast Cancer Res Treat, 2011. **126**(3): p. 771-8.

12. Teo, Z.L., et al., *Prevalence of PALB2 mutations in Australasian multiple-case breast cancer families.* Breast Cancer Res, 2013. **15**(1): p. R17.

13. Foo, T.K., et al., *Compromised BRCA1-PALB2 interaction is associated with breast cancer risk.* Oncogene, 2017. **36**(29): p. 4161-4170.

14. Blanco, A., et al., *Analysis of PALB2 gene in BRCA1/BRCA2 negative Spanish hereditary breast/ovarian cancer families with pancreatic cancer cases.* PLoS One, 2013. **8**(7): p. e67538.

15. Hellebrand, H., et al., *Germline mutations in the PALB2 gene are population specific and occur with low frequencies in familial breast cancer.* Hum Mutat, 2011. **32**(6): p. E2176-88.

16. Aoude, L.G., et al., *Assessment of PALB2 as a candidate melanoma susceptibility gene.* PLoS One, 2014. **9**(6): p. e100683.

17. Foulkes, W.D., et al., *Identification of a novel truncating PALB2 mutation and analysis of its contribution to early-onset breast cancer in French-Canadian women.* Breast Cancer Res, 2007. **9**(6): p. R83.

18. Dansonka-Mieszkowska, A., et al., *A novel germline PALB2 deletion in Polish breast and ovarian cancer patients.* BMC Med Genet, 2010. **11**: p. 20.

19. Blanco, A., et al., *Detection of a large rearrangement in PALB2 in Spanish breast cancer families with male breast cancer.* Breast Cancer Res Treat, 2012. **132**(1): p. 307-15.

20. Adank, M.A., et al., *Fanconi anemia gene mutations are not involved in sporadic Wilms tumor.* Pediatr Blood Cancer, 2010. **55**(4): p. 742-4.

21. Garcia, M.J., et al., *Analysis of FANCB and FANCN/PALB2 fanconi anemia genes in BRCA1/2-negative Spanish breast cancer families.* Breast Cancer Res Treat, 2009. **113**(3): p. 545-51.

22. Balia, C., et al., *PALB2: a novel inactivating mutation in a Italian breast cancer family.* Fam Cancer, 2010. **9**(4): p. 531-6.

23. Borecka, M., et al., *Mutation analysis of the PALB2 gene in unselected pancreatic cancer patients in the Czech Republic.* Cancer Genet, 2016. **209**(5): p. 199-204.

24. Caminsky, N.G., et al., *Prioritizing Variants in Complete Hereditary Breast and Ovarian Cancer Genes in Patients Lacking Known BRCA Mutations.* Hum Mutat, 2016. **37**(7): p. 640-52.

25. Catucci, I., et al., *PALB2 sequencing in Italian familial breast cancer cases reveals a high-risk mutation recurrent in the province of Bergamo.* Genet Med, 2014. **16**(9): p. 688-94.

26. Guenard, F., et al., *Evaluation of the contribution of the three breast cancer susceptibility genes CHEK2, STK11, and PALB2 in non-BRCA1/2 French Canadian families with high risk of breast cancer.* Genet Test Mol Biomarkers, 2010. **14**(4): p. 515-26.

27. Hofstatter, E.W., et al., *PALB2 mutations in familial breast and pancreatic cancer.* Fam Cancer, 2011. **10**(2): p. 225-31.

28. Kluska, A., et al., *PALB2 mutations in BRCA1/2-mutation negative breast and ovarian cancer patients from Poland.* BMC Med Genomics, 2017. **10**(1): p. 14.

29. Myszka, A., et al., *Targeted massively parallel sequencing characterises the mutation spectrum of PALB2 in breast and ovarian cancer cases from Poland and Ukraine.* Fam Cancer, 2018. **17**(3): p. 345-349.

30. Papi, L., et al., *A PALB2 germline mutation associated with hereditary breast cancer in Italy.* Fam Cancer, 2010. **9**(2): p. 181-5.

31. Sauty de Chalon, A., et al., *Are PALB2 mutations associated with increased risk of male breast cancer?* Breast Cancer Res Treat, 2010. **121**(1): p. 253-5.

32. Vietri, M.T., et al., *Analysis of PALB2 in a cohort of Italian breast cancer patients: identification of a novel PALB2 truncating mutation.* Fam Cancer, 2015. **14**(3): p. 341-8.

33. Zhen, D.B., et al., *BRCA1, BRCA2, PALB2, and CDKN2A mutations in familial pancreatic cancer: a PACGENE study.* Genet Med, 2015. **17**(7): p. 569-77.

34. Erkko, H., et al., *A recurrent mutation in PALB2 in Finnish cancer families.* Nature, 2007. **446**(7133): p. 316-9.

35. Kuusisto, K.M., et al., *Screening for BRCA1, BRCA2, CHEK2, PALB2, BRIP1, RAD50, and CDH1 mutations in high-risk Finnish BRCA1/2-founder mutation-negative breast and/or ovarian cancer individuals.* Breast Cancer Res, 2011. **13**(1): p. R20.

36. Mucaki, E.J., et al., *A unified analytic framework for prioritization of non-coding variants of uncertain significance in heritable breast and ovarian cancer.* BMC Med Genomics, 2016. **9**: p. 19.

37. Pakkanen, S., et al., *PALB2 variants in hereditary and unselected Finnish prostate cancer cases.* J Negat Results Biomed, 2009. **8**: p. 12.

38. Cecener, G., et al., *Association of PALB2 sequence variants with the risk of early-onset breast cancer in patients from Turkey.* Mol Biol Rep, 2016. **43**(11): p. 1273-1284.

39. Stafford, J.L., et al., *Reanalysis of BRCA1/2 negative high risk ovarian cancer patients reveals novel germline risk loci and insights into missing heritability.* PLoS One, 2017. **12**(6): p. e0178450.

40. Tung, N., et al., *Frequency of mutations in individuals with breast cancer referred for BRCA1 and BRCA2 testing using next-generation sequencing with a 25-gene panel.* Cancer, 2015. **121**(1): p. 25-33.

41. Shindo, K., et al., *Deleterious Germline Mutations in Patients With Apparently Sporadic Pancreatic Adenocarcinoma.* J Clin Oncol, 2017. **35**(30): p. 3382-3390.

42. Leyton, Y., et al., *Association of PALB2 sequence variants with the risk of familial and early-onset breast cancer in a South-American population.* BMC Cancer, 2015. **15**: p. 30.

43. Silvestri, V., et al., *PALB2 mutations in male breast cancer: a population-based study in Central Italy.* Breast Cancer Res Treat, 2010. **122**(1): p. 299-301.

44. Sluiter, M., S. Mew, and E.J. van Rensburg, *PALB2 sequence variants in young South African breast cancer patients.* Fam Cancer, 2009. **8**(4): p. 347-53.

45. Tischkowitz, M., et al., *Analysis of the gene coding for the BRCA2-interacting protein PALB2 in hereditary prostate cancer.* Prostate, 2008. **68**(6): p. 675-8.

46. Tischkowitz, M., et al., *Analysis of PALB2/FANCN-associated breast cancer families.* Proc Natl Acad Sci U S A, 2007. **104**(16): p. 6788-93.

47. Phuah, S.Y., et al., *Prevalence of PALB2 mutations in breast cancer patients in multi-ethnic Asian population in Malaysia and Singapore.* PLoS One, 2013. **8**(8): p. e73638.

48. Li, Y.T., et al., *PALB2 mutations in breast cancer patients from a multi-ethnic region in northwest China.* Eur J Med Res, 2015. **20**: p. 85.

49. Nakagomi, H., et al., *Analysis of PALB2 mutations in 155 Japanese patients with breast and/or ovarian cancer.* Int J Clin Oncol, 2016. **21**(2): p. 270-275.

50. Xie, Y., et al., *Mutation screening of 10 cancer susceptibility genes in unselected breast cancer patients.* Clin Genet, 2018. **93**(1): p. 41-51.

51. Yang, X.R., et al., *Prevalence and spectrum of germline rare variants in BRCA1/2 and PALB2 among breast cancer cases in Sarawak, Malaysia.* Breast Cancer Res Treat, 2017. **165**(3): p. 687-697.

52. Zhang, K., et al., *Germline mutations of PALB2 gene in a sequential series of Chinese patients with breast cancer.* Breast Cancer Res Treat, 2017. **166**(3): p. 865-873.

53. Cao, A.Y., et al., *The prevalence of PALB2 germline mutations in BRCA1/BRCA2 negative Chinese women with early onset breast cancer or affected relatives.* Breast Cancer Res Treat, 2009. **114**(3): p. 457-62.

54. Catucci, I., et al., *Germline mutations in BRIP1 and PALB2 in Jewish high cancer risk families.* Fam Cancer, 2012. **11**(3): p. 483-91.
