# Supplementary material for: A global functional analysis of missense mutations reveals two major hotspots in the PALB2 tumor suppressor
Source: Nucleic Acids Res. 2019 Oct 5;47(20):10662–77. doi: 10.1093/nar/gkz780 (PMC6847799; doi:10.1093/nar/gkz780)
Supplement: gkz780_Supplemental_Files [file gkz780_supplemental_files.zip › Table S2. In silico characterization of 44 PALB2 missense VUS.docx]

**Table S2. *In silico* characterization of 44 *PALB2* missense VUS**

| **Genomic position** | **Protein Change** | ***In silico* pathogenicity prediction** | | | | | |
| --- | --- | --- | --- | --- | --- | --- | --- |
|  |  | M-CAP^1^ | | VEST3.0^2^ | REVEL^3^ | | |
| Chr16:23652468 | p.P4L | Likely Benign (0.011) | Probably Neutral (0.279) | | | | Probably Neutral (0.044) |
| Chr16:23652456 | p.P8L | Likely Benign (0.012) | Probably Neutral (0.703) | | | | Probably Neutral (0.056) |
| Chr16:23649446 | p.K18R | ─ | Probably Neutral (0.368) | | | | Probably Neutral (0.181) |
| Chr16:23649416 | p.Y28C | Possibly Pathogenic (0.062) | Probably Disease Causing (0.015) | | | | Probably Neutral (0.224) |
| Chr16:23649409 | p.K30N | Likely Benign (0.022) | Probably Neutral (0.115) | | | | Probably Neutral (0.089) |
| Chr16:23649405 | p.L32V | Likely Benign (0.022) | Probably Neutral (0.082) | | | | Probably Neutral (0.150) |
| Chr16:23649395 | p.L35P | Possibly Pathogenic (0.101) | Probably Disease Causing (0.009) | | | | Probably Neutral (0.350) |
| Chr16:23649272 | p.R37H | Possibly Pathogenic (0.039) | Probably Disease Causing (0.035) | | | | Probably Neutral (0.158) |
| Chr16:23649246 | p.H46Y | Likely Benign (0.004) | Probably Neutral (0.364) | | | | Probably Neutral (0.050) |
| Chr16:23649188 | p.P65L | Likely Benign (0.010) | Probably Neutral (0.183) | | | | Probably Neutral (0.029) |
| Chr16:23647641 | p.I76V | Likely Benign (0.009) | Probably Neutral (0.407) | | | | Probably Neutral (0.042) |
| Chr16:23647635 | p.V78I | Likely Benign (0.014) | Probably Neutral (0.286) | | | | Probably Neutral (0.004) |
| Chr16:23647625 | p.K81R | Likely Benign (0.020) | Probably Neutral (0.355) | | | | Probably Neutral (0.025) |
| Chr16:23647587 | p.E94K | Likely Benign (0.010) | Probably Neutral (0.225) | | | | Probably Neutral (0.062) |
| Chr16:23647523 | p.G115V | Likely Benign (0.012) | Probably Neutral (0.549) | | | | Probably Neutral (0.042) |
| Chr16:23647496 | p.T124I | Likely Benign (0.006) | Probably Neutral (0.773) | | | | Probably Neutral (0.014) |
| Chr16:23647362 | p.L169I | Likely Benign (0.021) | Probably Neutral (0.3) | | | | Probably Neutral (0.065) |
| Chr16:23647247 | p.P207R | Likely Benign (0.023) | Probably Neutral (0.292) | | | | Probably Neutral (0.032) |
| Chr16:23647238 | p.P210L | ─ | Probably Neutral (0.407) | | | | Probably Neutral (0.096) |
| Chr16:23646918 | p.T317P | Likely Benign (0.022) | Probably Neutral (0.462) | | | | Probably Neutral (0.028) |
| Chr16:23646911 | p.S319Y | Likely Benign (0.018) | Probably Neutral (0.388) | | | | Probably Neutral (0.041) |
| Chr16:23637715 | p.P864S | Likely Benign (0.008) | Probably Neutral (0.251) | | | | Probably Neutral (0.062) |
| Chr16:23635370 | p.V932M | ─ | Probably Neutral (0.063) | | | | Probably Neutral (0.128) |
| Chr16:23635348 | p.L939W | Possibly Pathogenic (0.060) | Probably Disease Causing (0.008) | | | | Probably Neutral (0.359) |
| Chr16:23634446 | p.L947S | Possibly Pathogenic (0.086) | Probably Disease Causing (0.011) | | | | Probably Neutral (0.376) |
| Chr16:23634445 | p.L947F | Possibly Pathogenic (0.065) | Probably Disease Causing (0.023) | | | | Probably Neutral (0.330) |
| Chr16:23634421 | p.S955R | Likely Benign (0.008) | Probably Neutral (0.429) | | | | Probably Neutral (0.009) |
| Chr16:23634390 | p.I966V | Likely Benign (0.014) | Probably Neutral (0.129) | | | | Probably Neutral (0.073) |
| Chr16:23634293 | p.G998E | ─ | Probably Neutral (0.139) | | | | Probably Neutral (0.290) |
| Chr16:23632747 | p.A1017T | Possibly Pathogenic (0.026) | Probably Neutral (0.087) | | | | Probably Neutral (0.202) |
| Chr16:23632742 | p.E1018D | Possibly Pathogenic (0.030) | Probably Neutral (0.091) | | | | Probably Neutral (0.118) |
| Chr16:23632723 | p.A1025T | Likely Benign (0.020) | Probably Neutral (0.096) | | | Probably Neutral (0.128) | |
| Chr16:23632707 | p.T1030I | Possibly Pathogenic (0.059) | Probably Disease Causing (0.014) | | | Uncertain Significance (0.434) | |
| Chr16:23625404 | p.K1041T | Likely Benign (0.015) | Probably Neutral (0.072) | | | Probably Neutral (0.113) | |
| Chr16:23625398 | p.G1043A | Likely Benign (0.016) | Probably Disease Causing (0.022) | | | Probably Neutral (0.273) | |
| Chr16:23619312 | p.S1075G | Likely Benign (0.019) | Probably Neutral (0.09) | | | Probably Neutral (0.136) | |
| Chr16:23619284 | p.S1084L | Likely Benign (0.010) | Probably Neutral (0.745) | | | Probably Neutral (0.050) | |
| Chr16:23619257 | p.I1093T | Likely Benign (0.025) | Probably Disease Causing (0.016) | | | Probably Neutral (0.220) | |
| Chr16:23619229 | p.S1102R | Likely Benign (0.016) | Probably Neutral (0.058) | | | Probably Neutral (0.102) | |
| Chr16:23619221 | p.V1105A | Likely Benign (0.012) | Probably Disease Causing (0.029) | | | Probably Neutral (0.253) | |
| Chr16:23619193 | p.Q1114H | Likely Benign (0.006) | Probably Neutral (0.346) | | | Probably Neutral (0.068) | |
| Chr16:23614985 | p.L1119P | Possibly Pathogenic (0.129) | Probably Disease Causing (0.009) | | | Probably Disease Causing (0.465) | |
| Chr16:23614923 | p.W1140G | Possibly Pathogenic (0.140) | Probably Disease Causing (0.01) | | | Probably Disease Causing (0.563) | |
| Chr16:23614913 | p.L1143P | Likely Benign (0.017) | Probably Disease Causing (0.012) | | | Probably Neutral (0.245) | |
| Chr16:23614802 | p.I1180T | Possibly Pathogenic (0.041) | Probably Disease Causing (0.011) | | | Probably Neutral (0.337) | |

^1^ M-CAP prediction is only possible for variants with allele frequency ≤1% (Jagadeesh *et al*. 2016). ^2^ VEST scores range from 0 to 1, with 1 indicating a confident prediction of a functional mutation. VEST scores indicated in parenthesis are the predicted p-values for pathogenicity. We classified the variants as Probably Disease Causing when p ≤ 0.05 and as Probably Neutral when p > 0.05 (Carter *et al*. 2013). ^3^ REVEL scores range from 0 to 1, with higher scores reflecting greater likelihood of a disease-causing variant. When the scores are >0.450 the variants can be predicted more accurately as Probably Disease Causing. When scores are ≤ 0.400 the variants can be predicted more accurately as Probably Neutral. Uncertain significance refers to when scores are not able to differentiate between Probably Neutral and Probably Disease Causing (0.400-0.450) (Ioannidis *et al*. 2016). The scores for VEST and REVEL tools were classified as Probably Neutral, Probably Disease Causing and Uncertain significance by the authors, according to the predicted scores and each tool’s nomenclature.

Carter H. Douville C. Stenson PD. Cooper DN & Karchin R (2013) Identifying Mendelian disease genes with the Variant Effect Scoring Tool. *BMC Genomics*

Ioannidis NM. Rothstein JH. Pejaver V. Middha S. McDonnell SK. Baheti S. Musolf A. Li Q. Holzinger E. Karyadi D. Cannon-Albright LA. Teerlink CC. Stanford JL. Isaacs WB. Xu J. Cooney KA. Lange EM. Schleutker J. Carpten JD. Powell IJ. et al (2016) REVEL: An Ensemble Method for Predicting the Pathogenicity of Rare Missense Variants. *American Journal of Human Genetics*

Jagadeesh KA. Wenger AM. Berger MJ. Guturu H. Stenson PD. Cooper DN. Bernstein JA & Bejerano G (2016) M-CAP eliminates a majority of variants of uncertain significance in clinical exomes at high sensitivity. *Nature Genetics*
