# Supplementary figures and images for: A global functional analysis of missense mutations reveals two major hotspots in the PALB2 tumor suppressor
Source: Nucleic Acids Res. 2019 Oct 5;47(20):10662–77. doi: 10.1093/nar/gkz780 (PMC6847799; doi:10.1093/nar/gkz780)

A

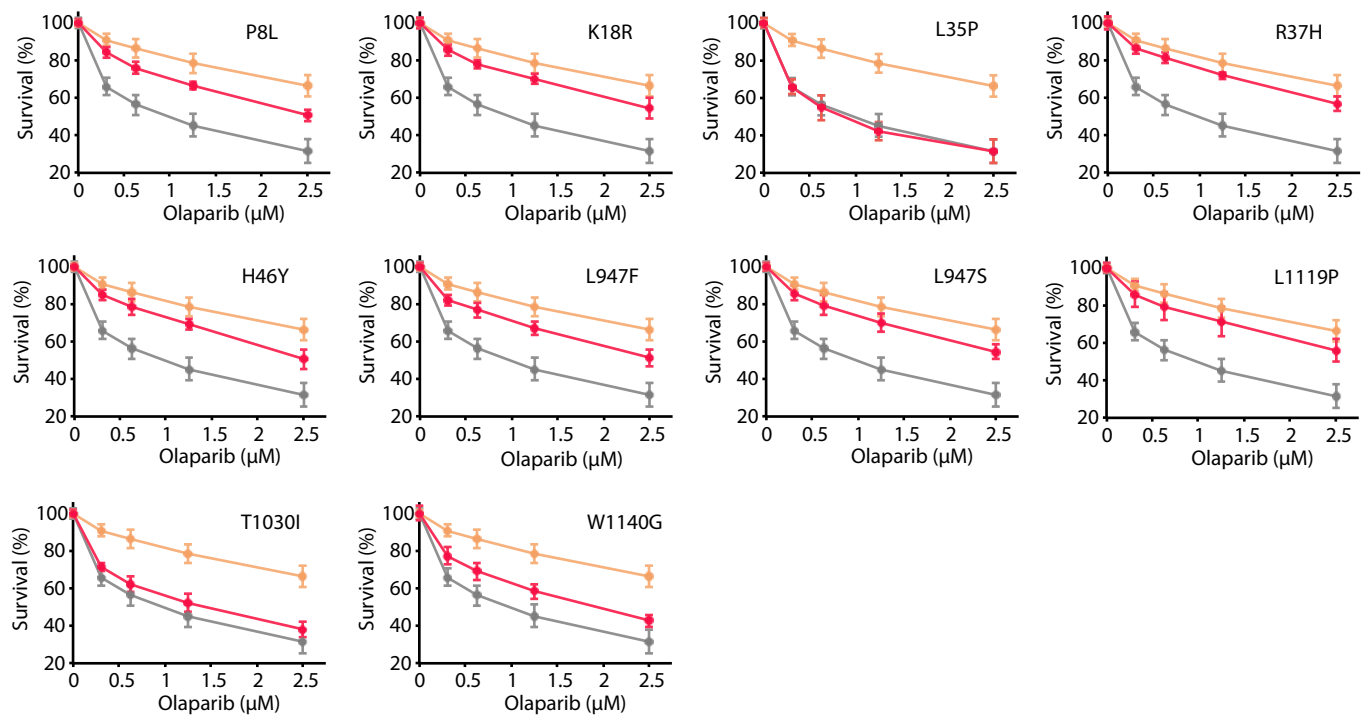

B

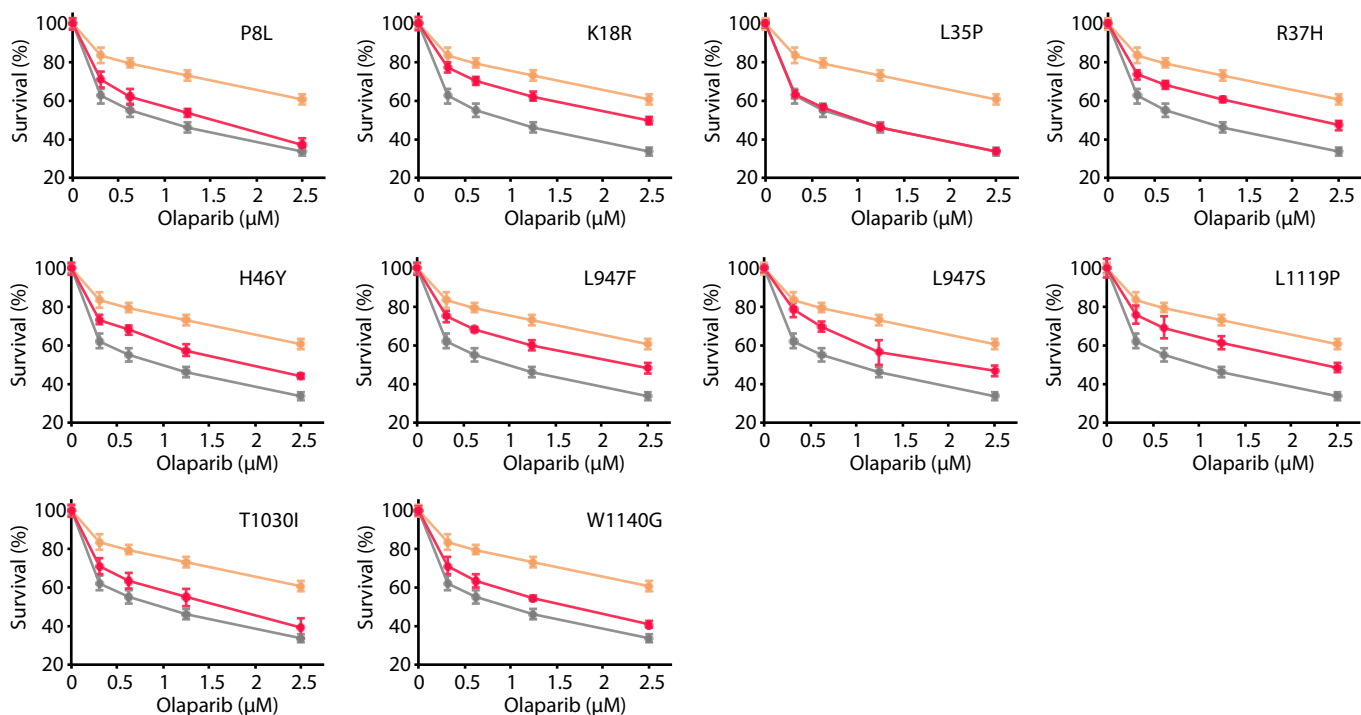

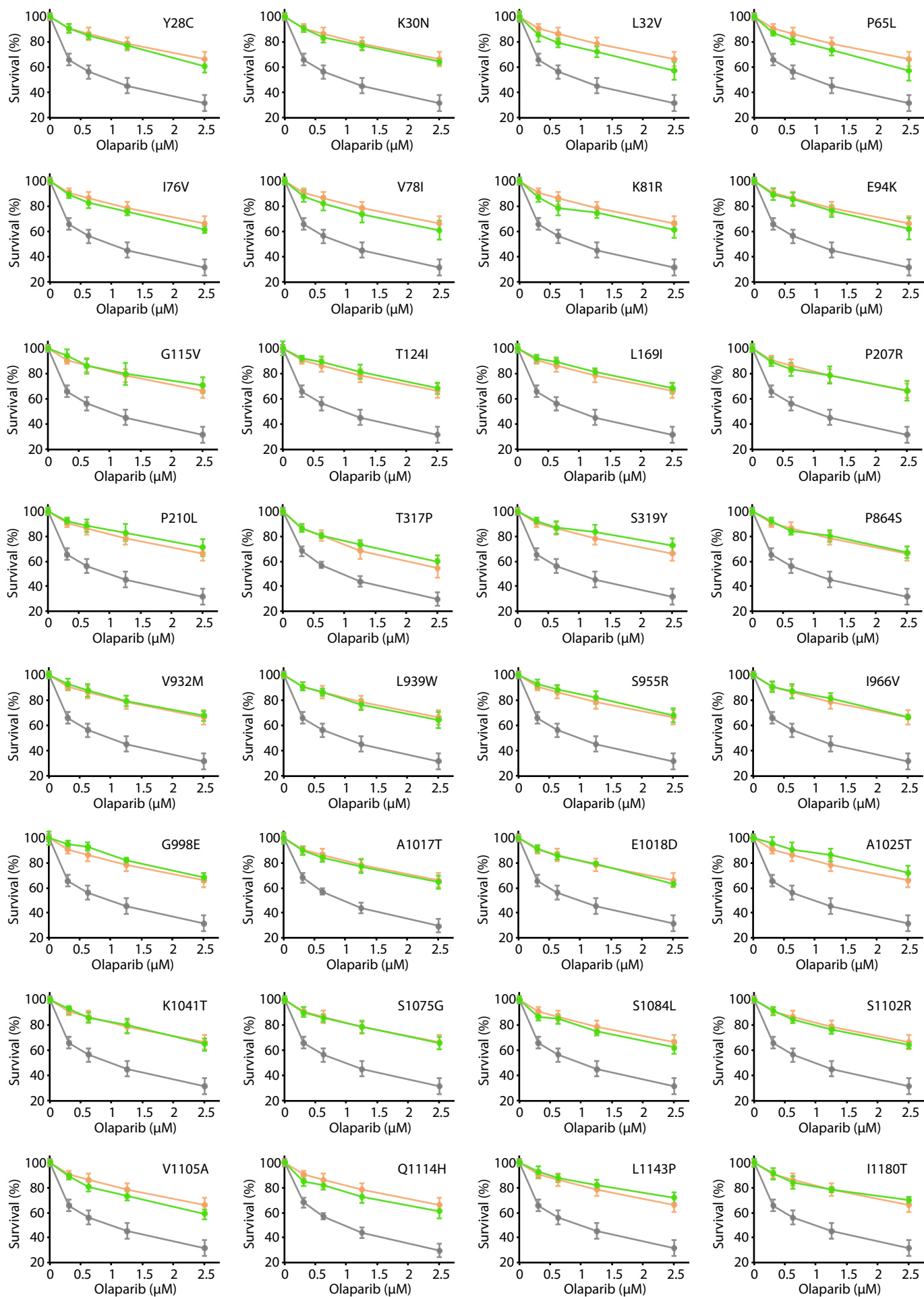

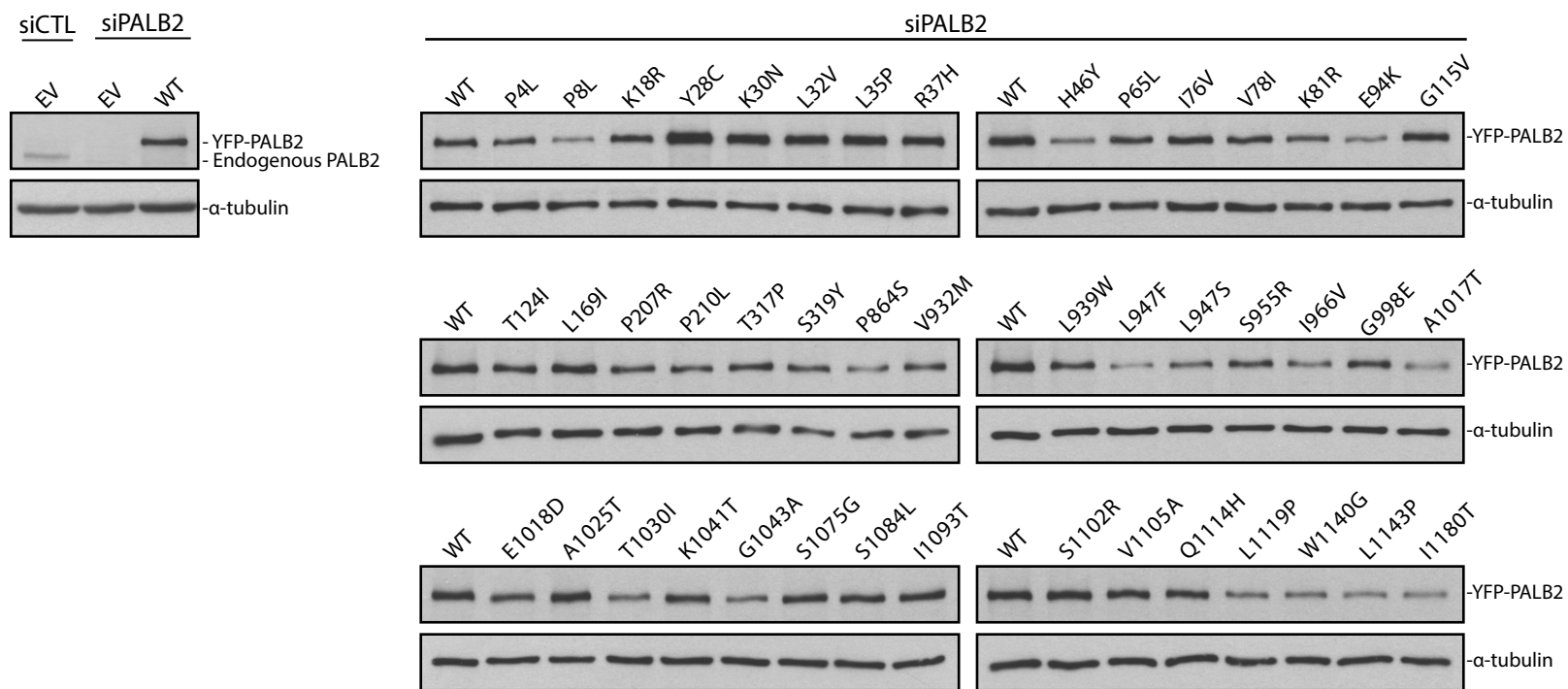

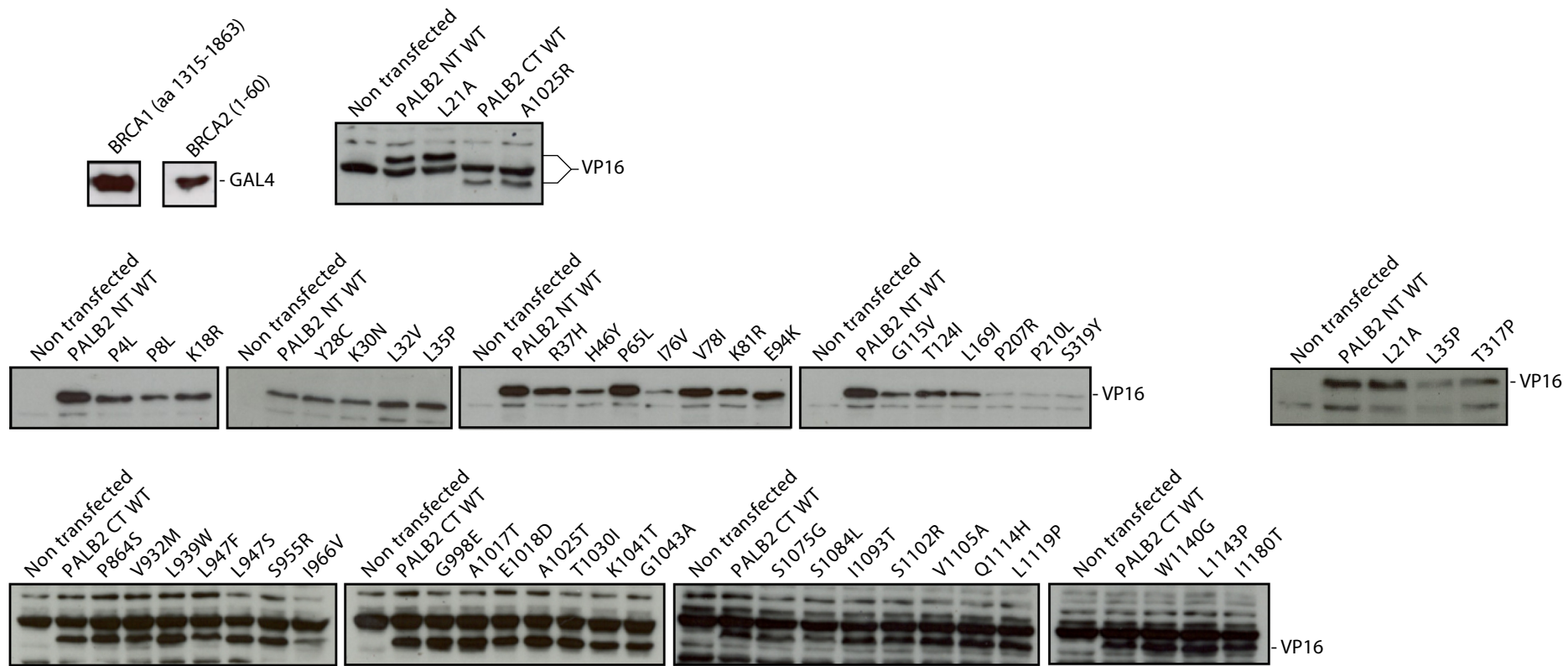

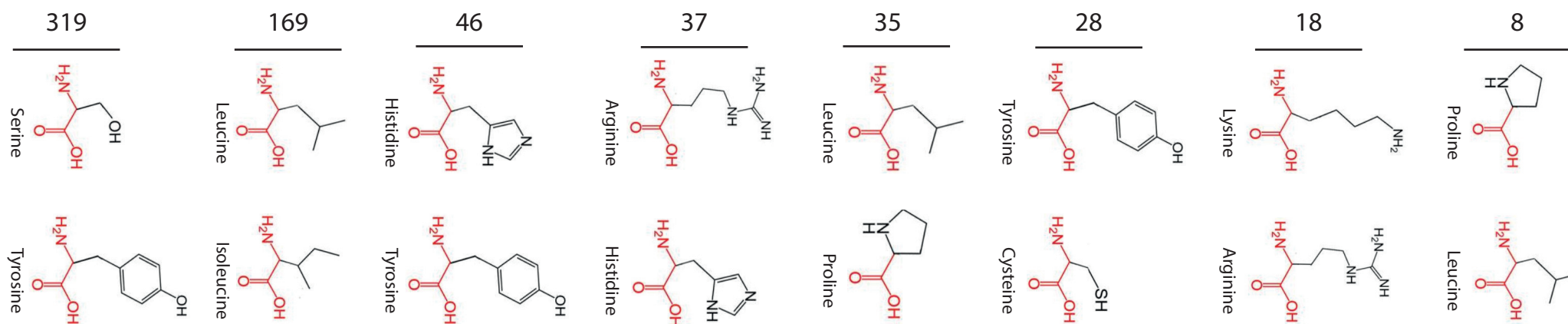

## Variants in the WD40 region

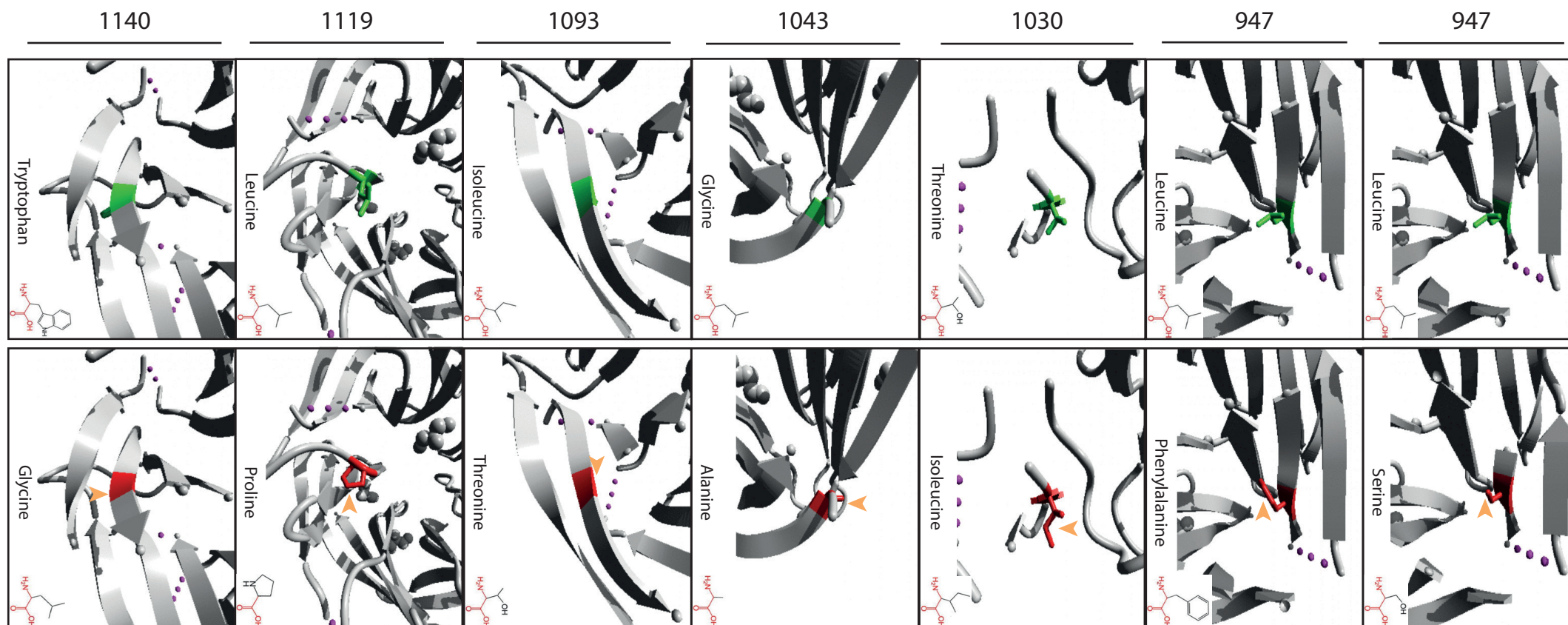

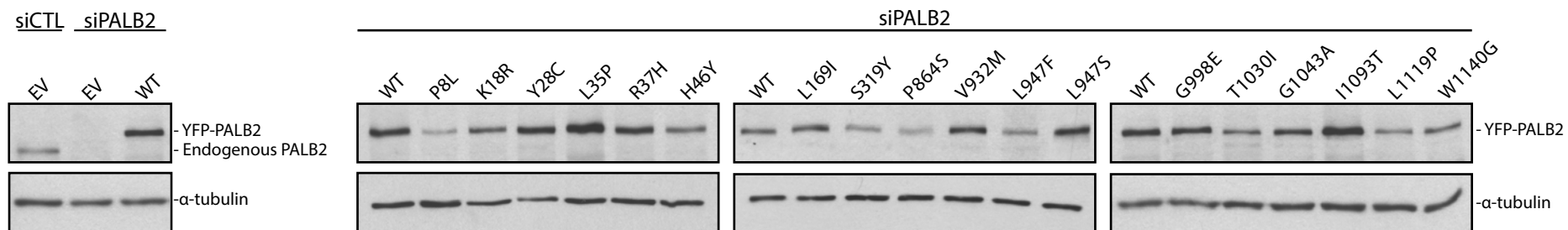

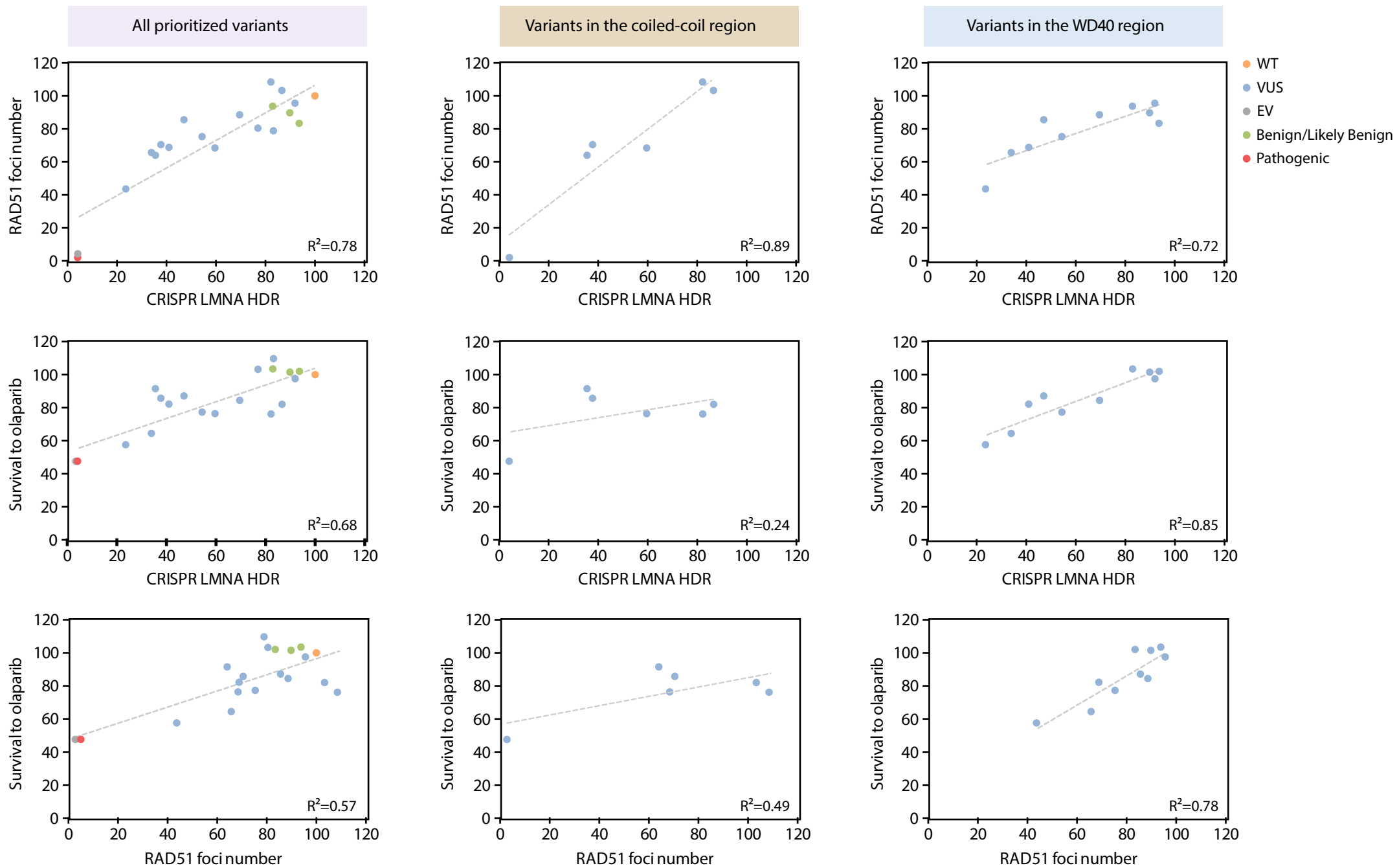

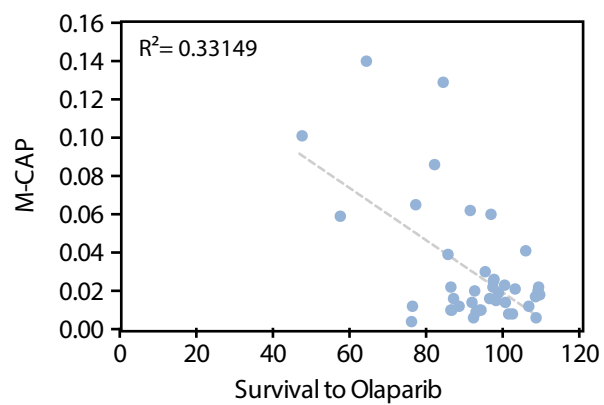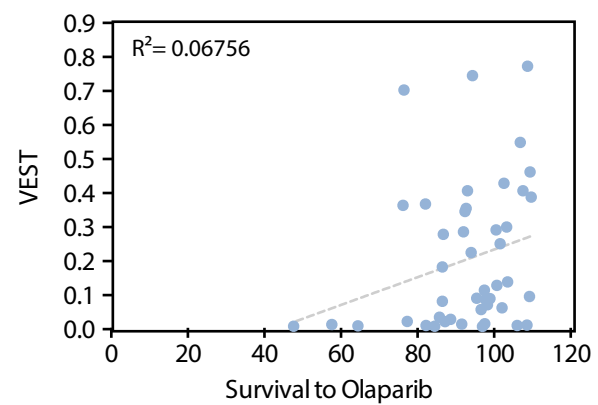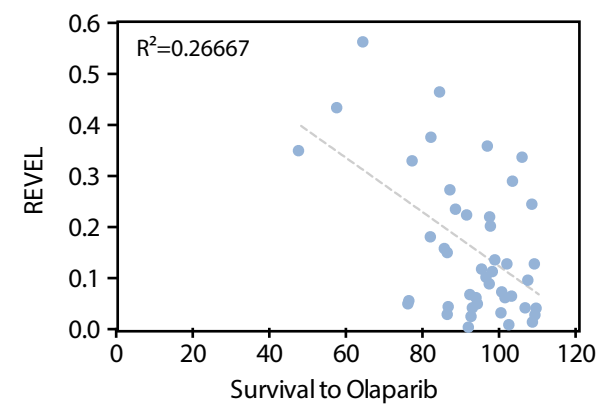

Supplement: gkz780_Supplemental_Files [file gkz780_supplemental_files.zip › Suppl._Figures_Rodrigue_et_al.--.pdf]
